# Systematic Evaluation of Imine‐Reducing Enzymes: Common Principles in Imine Reductases, β‐Hydroxy Acid Dehydrogenases, and Short‐Chain Dehydrogenases/ Reductases

**DOI:** 10.1002/cbic.202000213

**Published:** 2020-05-29

**Authors:** Peter Stockinger, Sebastian Roth, Michael Müller, Jürgen Pleiss

**Affiliations:** ^1^ Institute of Biochemistry and Technical Biochemistry University of Stuttgart Allmandring 31 70569 Stuttgart Germany; ^2^ Institute of Pharmaceutical Sciences Albert-Ludwigs-Universität Freiburg Albertstrasse 25 79104 Freiburg Germany

**Keywords:** beta-hydroxy acid dehydrogenases, flanking residues, imine reductases, intrinsic disorder, short-chain dehydrogenases/reductases

## Abstract

The enzymatic, asymmetric reduction of imines is catalyzed by imine reductases (IREDs), members of the short‐chain dehydrogenase/reductase (SDR) family, and β‐hydroxy acid dehydrogenase (βHAD) variants. Systematic evaluation of the structures and substrate‐binding sites of the three enzyme families has revealed four common principles for imine reduction: structurally conserved cofactor‐binding domains; tyrosine, aspartate, or glutamate as proton donor; at least four characteristic flanking residues that adapt the donor's p*K*_a_ and polarize the substrate; and a negative electrostatic potential in the substrate‐binding site to stabilize the transition state. As additional catalytically relevant positions, we propose alternative proton donors in IREDs and βHADs as well as proton relays in IREDs, βHADs, and SDRs. The functional role of flanking residues was experimentally confirmed by alanine scanning of the imine‐reducing SDR from *Zephyranthes treatiae*. Mutating the “gatekeeping” phenylalanine at standard position 200 resulted in a tenfold increase in imine‐reducing activity.

## Graphical abstract

**The enzymatic, asymmetric reduction** of imines to chiral amines is a valuable biocatalytic approach owing to the high enantioselectivity of the reductases. Imine reductases, short‐chain dehydrogenases/reductases, and β‐hydroxy acid dehydrogenases are known to catalyze this reaction. Our systematic evaluation of the structures and substrate‐binding sites of the three enzyme families has revealed four common principles for enzymatic imine reduction.

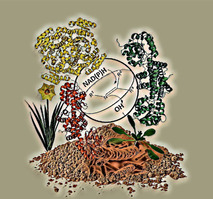

## Introduction

Chiral secondary amines are important building blocks for a broad range of pharmaceuticals, agrochemicals, and other specialty chemicals. Two promising biocatalytic routes are the reductive amination of ketones by reductive aminases (RedAms) or by amine dehydrogenases, and the asymmetric reduction of imines catalyzed by imine reductases (IREDs).^1^ Previously, a moderate sequence similarity and a high structural similarity between IREDs and β‐hydroxy acid dehydrogenases (βHADs) were uncovered.^2^ In both families, the monomers consist of an N‐terminal NADPH‐binding Rossmann‐like domain^3^ (domain 3.40.50.720 in the CATH database)^4^ and a C‐terminal helical domain. However, the active dimers are formed by domain swapping, with a long α8 helix in IREDs and a split α8 helix in βHADs.^5^ As a result, the substrate‐binding sites in IREDs consist of amino acids from different monomers, whereas they are built by the two domains of a single monomer in βHADs.^2^ The two enzyme families also show an overlap of catalytic activities: IREDs from *Streptosporangium roseumyy* (*R*‐IRED‐*Sr*) and *Paenibacillus elgii* not only catalyze the asymmetric reduction of imines, but also of the activated keto substrate 2,2,2‐trifluoroacetophenone.^6^ Conversely, the glyoxylate reductase from *Arabidopsis thaliana* is known to catalyze the reduction of different cyclic imine compounds.^7^ The similarity of the catalytic sites of IREDs and βHADs is supported by the observation that the exchange of a single amino acid in glyoxylate reductase from *A. thaliana* (sc‐βHAD‐*At*; K170D or K170F), γ‐hydroxybutyrate dehydrogenase from *Geobacter metallireducens* (sc‐βHAD‐*Gm*; K171D), and 6‐phosphogluconate dehydrogenase from *Lactococcus lactis subsp. cremoris* strain MG1363 (lc‐βHAD‐*Ll*; K184D) resulted in a significant decrease in the keto acid reduction activity and a further increase in the imine reduction activity. This increase was less striking for the βHAD K171D from *G. metallireducens*.^7^ Short‐chain dehydrogenases/reductases (SDRs) form a large protein family with high structural and functional divergence.^8^ They consist of a conserved N‐terminal NAD(P)H‐binding Rossmann‐like domain and a variable, substrate specificity determining C‐terminal region. Based on highly conserved sequence motifs, SDRs are classified into six subfamilies (classical, extended, intermediate, divergent, complex, and atypical SDRs).^9^, ^10^, ^11^, ^12^, ^13^, ^14^, ^15^ In addition to their catalytic activity toward carbonyl groups, some SDRs also show a moderate imine‐reducing activity. Noroxomaritidine reductase from *Narcissus pseudonarcissus* (SDR‐*Np*) catalyzes as a physiological reaction the reduction of the enone moiety in the plant alkaloid noroxomaritidine, but also shows a promiscuous catalytic activity toward the precursor imine norcraugsodine.^16^ Furthermore, SDR‐*Np* and a sequence‐homologous SDR from *Zephyranthes treatiae* (SDR‐*Zt*) display catalytic activity toward different imine compounds.^17^ Additionally, commercial glucose dehydrogenases are known to enantioselectively reduce bicyclic iminium compounds.^18^ Despite their promiscuous imine‐reducing activity, these members of the SDR family have no significant global sequence similarity to IREDs.^19^ Although the catalytic mechanism of IREDs is not completely understood,^20^ it is assumed that protonation of the imine occurs in the catalytic site.^21^ The resulting iminium is then reduced by a hydride transferred from NAD(P)H.^22^ Two superfamilies have been distinguished: the *R*‐ and the *S*‐selective IREDs. They differ in their catalytic machinery with a conserved aspartic acid or a tyrosine, respectively, as proton donor.^23^ However, as the orientation of the substrate depends critically on its structure, *R*‐ and *S*‐selective imine reductases convert some imines into *S* and *R* amines, respectively.^20^, ^24^ Therefore, IREDs with aspartate or glutamate as donor were assigned as D‐type, with tyrosine as Y‐type.^21^ The catalytic mechanism of βHADs is well understood. The reduction of the C=O group is initiated via protonation by a conserved lysine as proton donor, followed by hydride transfer from NADPH.^25^ Their catalytic mechanism is similar to the extensively studied mechanism of classical SDRs, which is mediated by a conserved catalytic triad or tetrad Lys‐Tyr‐(Asn)‐Ser. The tyrosine serves as a proton donor, and its p*K*_a_ is adjusted by interaction with the catalytic lysine. A water molecule can be bound between the catalytic lysine and the optional asparagine contributing to the proton relay system.^12^ The polarization of the substrate's carbonyl group is mediated by the catalytic serine.^12^ All three enzyme families (βHADs, SDRs, IREDs) show conformational changes upon cofactor and substrate binding. For the IRED from *Amycolatopsis orientalis* (*R‐*IRED*‐Ao*), three different conformations have been observed: the open apo form, the closed NADPH complex, and a complex with cofactor and product. In the closed complex, the volume of the substrate‐binding site considerably decreased.^24^ A comparable mechanism of induced fit has also been observed for βHADs and SDRs.^16^, ^26^, ^27^ Although sequence‐based classifications into distinct families provide a powerful tool, rigorous functional classification approaches could bias the selection of engineering templates. Despite of the separate family affiliation, we hypothesized the existence of common principles for imine‐reducing enzymes.

## Results

**Figure 1:**
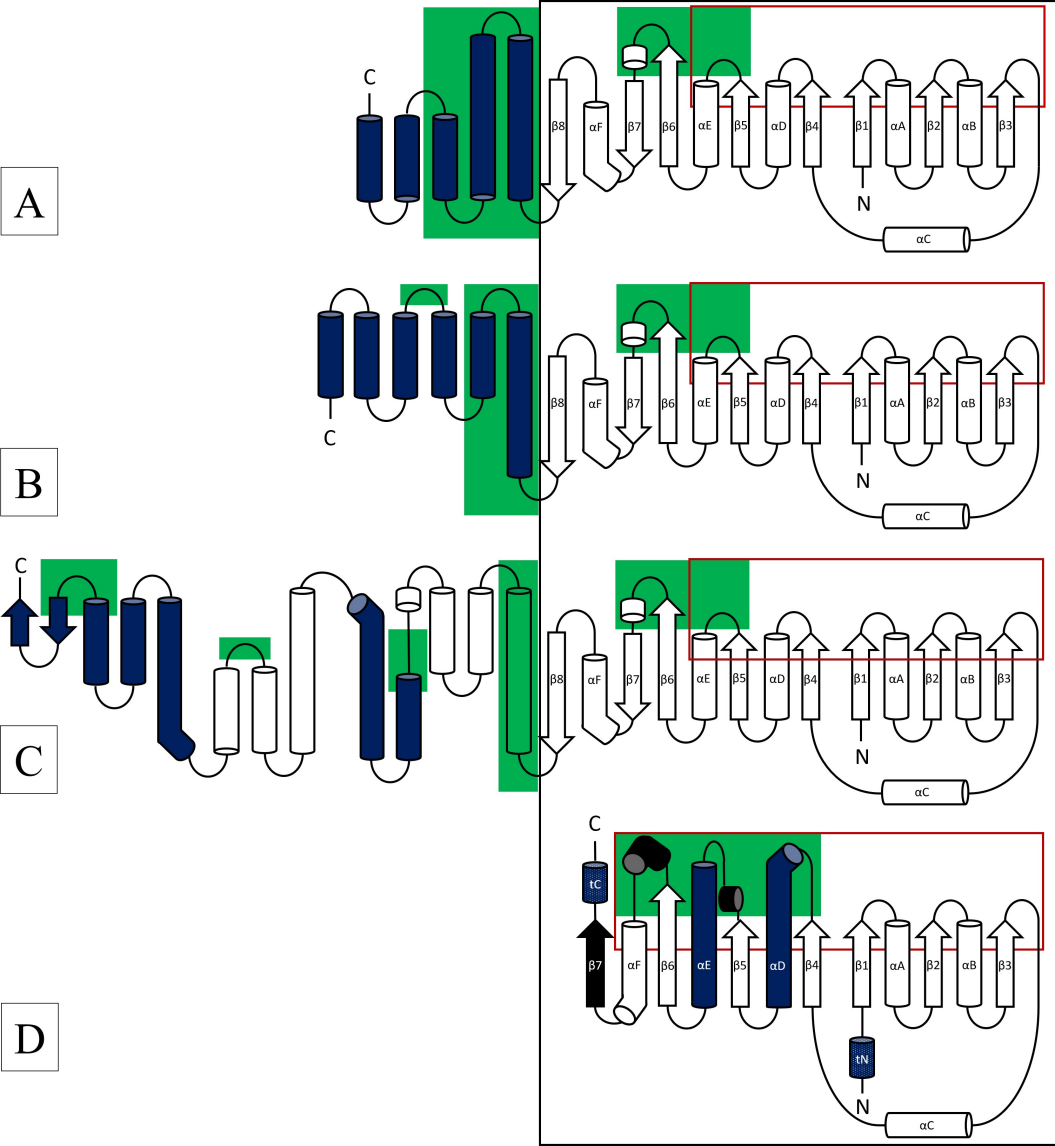
Structural scheme of A) IREDs, B) “short‐chain” βHADs, C) “long‐chain” βHADs, and D) classical SDRs. The black box marks the Rossmann‐like NADPH‐binding domains which were superimposed; the red boxes mark the secondary structures involved in cofactor binding. Blue coloring indicates the structures that are involved in multimerization, and the green areas indicate substrate‐interacting regions. In (D), secondary structures of the Rossmann‐like domain that were not superimposable with those of IREDs/βHADs are colored in black, and terminal helices that only appear in the tetrameric SDR are labeled (tN and tC).

Due to the difference in length of sc‐βHAD‐*At* and lc‐βHAD‐*Ll*, the relative orientation of the Rossmann‐like domains of lc‐βHAD‐*Ll* is twisted by 45°, and the dimerization is mediated by different helical regions (Figure S4B, C in the Supporting Information). In the dimeric SDR‐*Bv*, in the absence of a C‐terminal helical domain the contact is mediated by the two long helices of the Rossmann‐like domain of each monomer (Figure S4D). In the tetrameric SDR‐*Np*, two of these dimers associate along the axis of the β‐sheets (Figure S5). The Rossmann‐like domains of *R*‐IRED‐*Sr*, *R*‐IRED‐*Ao*, βHAD‐*At,* βHAD‐*Ll*, SDR‐*Bv*, and SDR‐*Np* were superimposed (Figure S6), which revealed a similar binding conformation of NADPH (Figure S7) and a similar location of the NADPH‐binding residues in the structurally conserved Rossmann fold (β‐strands β1–β5 and α‐helices αA–αE; Figure 1). IREDs and βHADs exhibit a classical Rossmann fold consisting of six parallel β‐strands (β1–β6) and an additional structural motif (β7‐αF‐β8) connecting the Rossmann fold to a helical domain. β6 and β7 are connected by a short α‐helix, and β7 and β8 by the curved α‐helix αF; thus, the β‐sheet of the Rossmann fold is extended by the two antiparallel β‐strands β7 and β8 (Figure 1A–C). Classical SDRs show a very similar N‐terminal half of the Rossmann‐like domain, but the C‐terminal half deviates (Figure 1D). In contrast to IREDs and βHADs, classical SDRs have additional short α‐helices after β5 and β6 and an additional seventh parallel β‐strand. Helices αD and αE are significantly longer than those in IREDs or βHADs, and helices αD and αF are kinked. Helix αF is a structural analogue in the three families, contributing to the Rossmann fold in SDRs and to the β7‐αF‐β8 motif in IREDs and βHADs. In comparison to the dimeric classical SDR‐*Bv*, the tetrameric SDR‐*Np* has additional N‐ and C‐terminal helices (tN and tC) which contribute to the contact surface between the two dimers. Whereas the NADPH‐binding site is structurally highly conserved in the three enzyme families, the substrate‐binding sites differ. The substrate‐binding sites in IREDs and in βHADs are formed by the connecting loops between β5 and αE and between β6 and β7, and by residues located on the N‐terminal helical domain (Figure 1). The substrate‐binding sites in classical SDRs, however, are formed by the short α‐helices after β5 and β6, and residues on the two long α‐helices αE and αD. In the dimeric SDR‐*Bv*, the loop after β5 is significantly shorter and does not display the helix observed in the imine‐reducing SDR‐*Np* (Figure S8). Additionally, the electrostatics of the substrate‐binding sites differ: the investigated IREDs display a negatively charged substrate‐binding site, whereas the investigated βHADs and SDRs have a mainly positively charged substrate‐binding site (Figure S9). However, SDR‐*Np* and SDR‐*Zt* exhibit a negatively charged residue (E212, classical SDR standard position 210) on the flexible helix involved in substrate binding. Furthermore, the mutation K184D in lc‐βHAD‐*Ll* leads to a negative charge around the catalytic site (Figure S10a). In contrast, in the less active sc‐βHAD‐*Gm* K171D, the substrate‐binding site remains positively charged (Figure S10b). For a detailed comparison of the substrate binding site residues, please see the Supporting Information.

**Figure 2:**
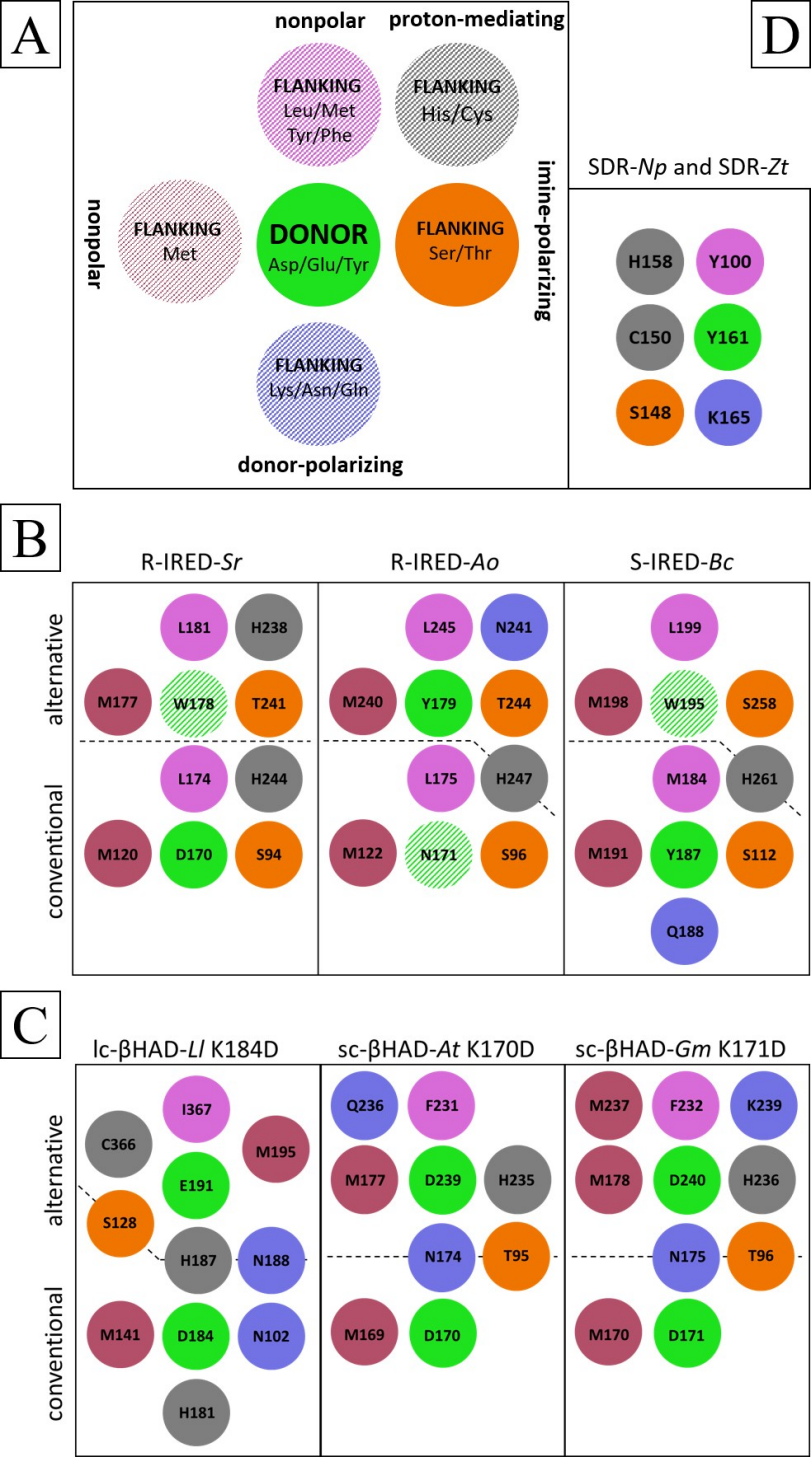
A) General scheme of imine‐reducing catalytic sites. Dashed coloring shows flanking residues that are not strictly present in all considered enzymes. The proton donor (green) is always flanked by one putatively imine‐polarizing amino acid (orange). Except for the conventional βHADs, all proton donors are flanked by at least one nonpolar amino acid (pink). One or more donor‐polarizing amino acids (blue) are present in all considered enzymes except *R*‐IRED‐*Sr*. The characteristic flanking methionine (red) was not observed in the imine‐reducing SDRs. Optionally, proton‐mediating residues flanking the imine‐polarizing residue can be present (gray). The proposed conventional and catalytic sites are schematically shown for the considered IREDs (B) and βHADs (C), where the dashed lines mark the respective affiliation of the flanking residues and dashed green coloring represents a non‐proton‐donating residue at the proposed alternative proton donor position. D) The considered imine‐reducing classical SDRs did not present the characteristic flanking methionine or an alternative catalytic site.

We hypothesize that all investigated catalytic sites consist of a proton donor (aspartate, glutamate, or tyrosine) and at least three flanking residues supporting the proton donation (Figure 2, Table S7). In addition to a nonpolar flanking residue which occurs in the considered IREDs, βHADs, and SDRs, one characteristic flanking methionine occurs in IREDs and βHADs. Except for *R*‐IRED‐*Sr*, all enzymes display at least one putatively donor‐polarizing residue. A serine or threonine flanking the proton donor occurs in all considered IREDs (IRED standard position 111; involved in cofactor binding),^2^ βHADs, and SDRs (classical SDR standard position 144). In the case of keto‐reducing SDRs, this catalytic serine has been proposed to polarize the carbonyl moiety.^11^, ^12^ Therefore, we hypothesize that these residues also polarize the imine moiety in the course of imine reduction, supplemented by optional proton‐mediating amino acids presumably forming a proton relay, as already proposed for βHADs^7^ and SDRs.^11^, ^12^ The exact composition and arrangement of these residues in the putative catalytic sites varies between the enzyme families and even among the family members. Additionally, we hypothesize the existence of alternative catalytic sites in IREDs and βHADs. Thereby, single residues can flank the conventional and the alternative proton donors simultaneously.

In *R*‐IRED‐*Sr*, amino acid D170 (IRED standard position 187) is the proton donor and is flanked by the imine‐polarizing S94, the nonpolar M120 and L174, and the proton‐mediating H244 (IRED standard positions 111, 137, 191, 261). Interestingly, amino acid W178 (IRED standard position 195) is flanked by the imine‐polarizing T241 (IRED standard position 258), the nonpolar M177 (IRED standard position 194) and L181 (IRED standard position 198), and the proton‐mediating H238 (IRED standard position 255; Figures 2B and S11B). In *R*‐IRED‐*Ao*, amino acid N171 (IRED standard position 187) at the conventional proton donor position is flanked by nonpolar M122 and L175 (IRED standard positions 137, 191). Compared to imine‐polarizing S94 and proton‐mediating H244 in *R*‐IRED‐*Sr*, residues S96 and H247 (IRED standard positions 111, 261) are not oriented toward the conventional proton donor position. On the basis of the published product complex,^24^ amino acid Y179 (IRED standard position 195) is suggested as the alternative proton donor (Figure S11A) flanked by the imine‐polarizing T244 (IRED standard position 258), the nonpolar M240 (IRED standard position 254) and L245 (IRED standard position 259), the donor‐polarizing N241 (IRED standard position 255), and the proton‐mediating H247 (IRED standard position 261; Figures 2B and S11C). In the *S*‐selective IRED from *Bacillus cereus* (*S*‐IRED‐*Bc*), amino acid Y187 (IRED standard position 187) is the conventional proton donor and is flanked by the imine‐polarizing S112 (IRED standard position 111), the nonpolar M184 and M191 (IRED standard positions 184, 191), the donor‐polarizing Q188 (IRED standard position 188), and the proton‐mediating H261 (IRED standard position 261). W195 at the alternative donor position (IRED standard position 195) is probably flanked by the imine‐polarizing S258 (IRED standard position 258) and the nonpolar M198 (IRED standard position 198) and L199 (IRED standard position 199; Figures 2B and S11D). The lack of a complexed crystal structure gives rise to uncertainties. In lc‐βHAD‐*Ll*, amino acid K184 is the conventional proton donor and is flanked by the imine‐polarizing S128, the nonpolar M141, the proton‐mediating H181 and H187, and the donor‐polarizing N102 and N188. In the lc‐βHAD‐*Ll* variant with improved imine‐reducing activity, K184 is replaced by D184. The amino acid E191 is proposed as the alternative proton donor and is flanked by the imine‐polarizing S128, the nonpolar M195 and I367, the proton‐mediating H187 and C366, and the donor‐polarizing N188 (Figures 2C and S12C). In sc‐βHAD‐*At*, amino acid K170 is the conventional proton donor that is flanked by the imine‐polarizing T95, the nonpolar M169, and the donor‐polarizing N174. In the sc‐βHAD‐*At* variant with improved imine‐reducing activity, K170 is replaced by D170. The amino acid D239 is proposed as the alternative proton donor and is flanked by the imine‐polarizing T95, the nonpolar M177 and F231, the proton‐mediating H235, and the donor‐polarizing N174 and Q236 (Figures 2C and S12D). In sc‐βHAD‐*Gm*, amino acid K171 is the conventional proton donor and is flanked by the imine‐polarizing T96, the nonpolar M170, and the donor‐polarizing N175. In the sc‐βHAD‐*Gm* variant with slightly improved imine‐reducing activity, K171 is replaced by D171. The amino acid D240 is proposed as the alternative proton donor and is flanked by the imine‐polarizing T96, the nonpolar M178, F232 and M237, the proton‐mediating H236, and the donor‐polarizing N175, and K239 (Figures 2C and S12E). In SDR‐*Np* and SDR‐*Zt*, Y161 (classical SDR standard position 159) is the conventional proton donor and is flanked by the imine‐polarizing S148 (classical SDR standard position 144), the nonpolar Y100 (classical SDR standard position 96), the proton‐mediating C150 and H158 (classical SDR standard positions 146, 156), and the conventional donor‐polarizing K165 (classical SDR standard position 163; Figures 2D and S13). No flanking methionine was found in the imine‐reducing SDRs.

**Figure 3:**
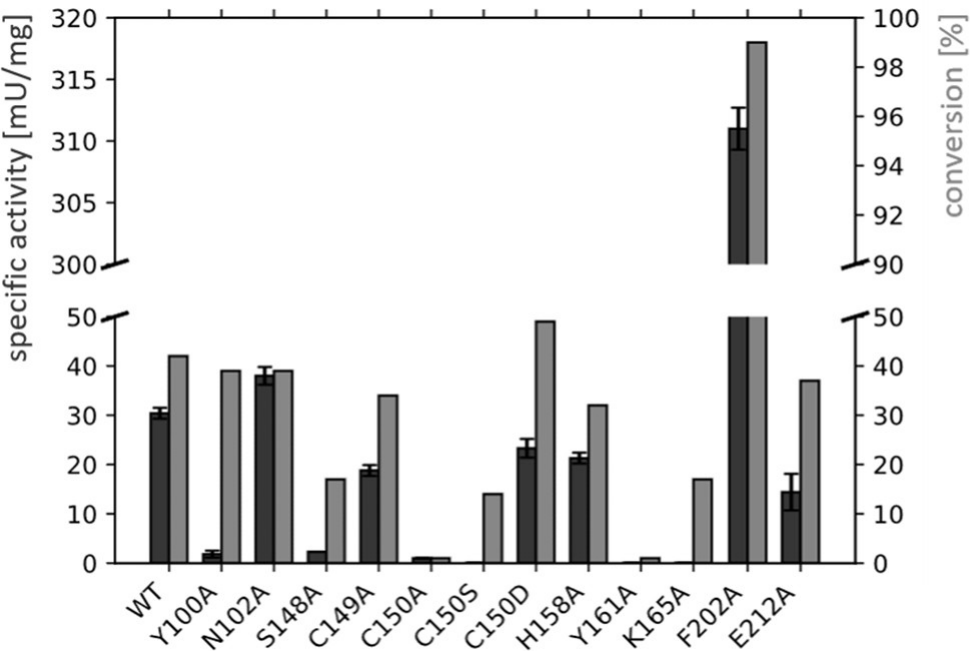
Biotransformation results for wild‐type, alanine variants, and C150S+C150D variants of SDR‐*Zt*. The specific activity (dark gray) on TMI and its conversion after reaction for 3 h (light gray) are shown. Due to the significantly increased activity of variant F202A, a scaling interruption has been introduced for visualization purposes.

To evaluate the functional relevance of residues in the substrate‐binding site for imine reduction, alanine variants of selected residues of SDR‐*Zt* were generated. In addition to the conventional catalytic residues for keto reduction, S148, Y161, and K165 (classical SDR standard positions S144, Y159, K163), substrate‐binding residues with rare occurrence (<4 %), Y100, N102, C149, C150, H158, F202, and E212 (classical SDR standard positions 96, 98, 145, 146, 156, 200, 210), were mutated (Figure S14). Using 1 and 10 mM 2,3,3‐trimethylindolenine (TMI) as model imine substrate, respectively, specific activity and conversion after 3 hours were determined (Table S8, Figure 3). The alanine variants of the conventional catalytic amino acids and of two proposed flanking residues (Y100, C150) nearly lost their activity toward TMI. For variants C150A and Y161A, the substrate conversion was low (in both cases <1 %) and therefore consistent with the respective specific activity. However, a discrepancy was noticed for Y100A, S148A, and K165A, for which conversions of 39 %, 17 %, and 17 % were found, respectively, whereas poor specific activities (2±1 mU/mg, 2±0.1 mU/mg, and 0 mU/mg) were detected. This deviation could be caused by different KM values or substrate inhibition of the variants, as the two screening methods required different substrate concentrations. While SDR‐*Zt* variants Y100A and K165A converted TMI with high enantioselectivity (>98 % *ee* of *R* product), variant S148A displayed a loss in enantioselectivity (17 % *ee* of *R* product). The alanine variant of the flanking histidine (H158A) exhibited a decreased activity (32 % conversion, 21±1 mU/mg specific activity), similar to C149A (34 % conversion, 19±1 mU/mg specific activity) and E212A (37 % conversion, 14±4 mU/mg).

While variant N102A showed a slightly increased specific activity of 38±2 mU/mg, the conversion (39 %) was roughly similar to that of the wild‐type SDR‐*Zt* (42 % conversion, 30±1 mU/mg). Interestingly, the F202A mutation resulted in a 10‐fold increase in activity (311±2 mU/mg) compared to the wild type, whereby >99 % of TMI was converted. To explore whether C150 serves as a flanking residue which is essential for imine‐reducing activity, the variants C150S and C150D were experimentally investigated (Table S8, Figure 3). The exchange against the less acidic serine (variant C150S) resulted in a significantly decreased activity (14 % conversion, undetectable specific activity) compared to wild‐type SDR‐*Zt*, whereas the exchange against the acidic aspartate (variant C150D) led to a comparable specific activity (23±2 mU/mg) and 49 % conversion of TMI.

## Discussion

For the access to chiral amines, nature has evolved different protein scaffolds into a broad range of biocatalysts with different substrate specificities and high enantioselectivities of up to >99 %.^21^, ^23^, ^24^ IREDs and βHADs are evolutionary related, which is reflected in their similar structure and sequence.^2^, ^21^ SDRs display a different structure and no sequence similarity to IREDs and βHADs.^2^, ^19^, ^21^ Nevertheless, certain SDRs also catalyze the reduction of C=N bonds.^17^, ^18^ The occurrence of this activity in different enzyme families suggests an analogous development of imine‐reducing machineries with a similar catalytic mechanism, namely the transfer of a proton and a hydride to the respective substrate. Guided by the enzyme mechanism of C=O reduction and the systematic evaluation of sequences and structures of IREDs, βHADs, and imine‐reducing SDRs, 12 experimentally characterized and three published variants,^7^ we have identified four common principles enabling imine‐reducing activity in NAD(P)H‐dependent enzymes: 1) a suitable cofactor‐binding domain, 2) proton donors adjusted in their p*K*_a_, 3) flanking residues which contribute to p*K*_a_ adjustment, and 4) a negative electrostatic potential in the substrate‐binding site.

In the investigated enzyme families, the cofactor‐binding site is provided by the ubiquitous Rossmann‐like domain, which enables a productive orientation of the cofactor to allow effective hydride donation. Sequence motifs for the binding of these cofactors need to be present.^28^, ^29^ Additionally, residues that are involved in cofactor binding can influence the ratio between oxidative and reductive activity,^30^ or contribute to local secondary structures and conformational changes.^31^ Although all investigated enzymes utilize NADPH as cofactor, other hydride donors are conceivable, such as NADH which serves as a cofactor for IREDs.^32^, ^33^

Next to aspartates, glutamates and tyrosines as proton donor, it cannot be excluded that protonation occurs with other residues (e. g., histidines). A p*K*_a_ adjustment of the respective donor is required to enable proton transfer under experimental conditions (pH 6–8).^7^, ^17^, ^21^, ^24^ Nonpolar flanking residues or negatively charged residues in the neighborhood result in an increased p*K*_a_ of the proton donor, whereas polar flanking residues result in a decreased p*K*_a_.

In D‐type IREDs, the p*K*_a_ adjustment is enabled by two nonpolar flanking residues. Grogan and co‐workers proposed that two nonpolar flanking residues in *R*‐IRED‐*Sk* enable the strongly acidic proton donor to mediate proton donation.^5^ This is supported by the p*K*_a_ of 7.7 of proton donor D170 at the highly conserved conventional donor position (IRED standard position 187; 93 % D in *R*‐IREDs). Remarkably, the nonpolar flanking residues M120 (IRED standard position 137) and L174 (IRED standard position 191) are highly conserved (94 % M and 96 % L, respectively) in *R*‐IREDs. Apart from H244 (IRED standard position 261; 31 % H in *R*‐IREDs) as proton‐mediating flanking residue and S94 (IRED standard position 111; 45 % T, 33 % S in *R*‐IREDs) as imine‐polarizing flanking residue, no polar flanking residues were observed.

In Y‐type IREDs (IRED standard position 187; 100 % Y in *S*‐IREDs), the p*K*_a_ adjustment seems to be enabled by a donor‐polarizing flanking residue. In contrast to D‐type IREDs that feature two nonpolar flanking residues, Y‐type feature one nonpolar (IRED standard position 191; 51 % L, 39 % M in *S*‐IREDs) and one predominantly polar flanking residue (IRED standard position 184; 56 % Q, 36 % A, 1 % L, 5 % M in *S*‐IREDs). This supports the hypothesis of the importance of suitable flanking residues to adapt the p*K*_a_ of the respective proton donor.

Recently, some exceptional IREDs lacking the conventional proton donors have been identified.^24^, ^34^ For instance, *R*‐IRED‐*Ao* lacks the conserved aspartate at IRED standard position 187. Therefore, the conventional catalytic site seems to be “inactive” while the alternative catalytic site could be considered as “active”, as it displays a tyrosine at the alternative donor position (IRED standard position 195; 57 % Y in *R*‐IREDs). As in Y‐type IREDs, the p*K*_a_ adjustment seems to be enabled by a donor‐polarizing flanking residue: N241 (IRED standard position 255; 49 % Q, 14 % H, 12 % N in *R*‐IREDs).

In the investigated K→D variants of βHADs,^7^ the p*K*_a_ adjustment is enabled by at least one donor‐polarizing flanking residue connecting the engineered conventional proton donor (D184 in lc‐βHAD‐*Ll*; D170 in sc‐βHAD‐*At*; D171 in sc‐βHAD‐*Gm*) with the alternative proton donor (E191 in lc‐βHAD‐*Ll*; D239 in sc‐βHAD‐*At*; D240 in sc‐βHAD‐*Gm*). Such neighboring carboxylates are assumed to result in a higher p*K*_a_ value^35^ and therefore enable the proton transfer to the imine substrate under experimental conditions. Additionally, conventional and alternative catalytic sites display a methionine as nonpolar flanking residue. In sc‐βHAD‐*At* and sc‐βHAD‐*Gm*, M169 and M170, respectively, could serve as nonpolar flanking residue of the respective conventional proton donor, although their side chains are oriented away from the proton donor in the crystal structures. However, upon substrate binding, they might reorient toward the proton donor, providing a similar function as M141 in lc‐βHAD‐*Ll*.

In imine‐reducing SDRs, the p*K*_a_ adjustment of the proton donor tyrosine (classical SDR standard position 159) is presumably realized by lysine (classical SDR standard position 163) as donor‐polarizing flanking residue, which also adjusts the p*K*_a_ of the proton donor to enable keto reduction.^8^, ^11^, ^12^, ^15^, ^36^ However, due to its positive charge, the electrostatic potential in the substrate‐binding site is detrimental for stabilization of the positively charged iminium transition state. This might be partially compensated by the acidic character of the proton‐mediating flanking C150 (classical SDR standard position 146), which is supported by the reduced specific activity of the C150A and C150S variants of SDR‐*Zt*, whereas the C150D variant exhibits comparable activity to the wild type. In addition to the local electrostatic effect of the flanking residues on the proton donor, the overall electrostatic potential in the substrate‐binding site of imine‐reducing enzymes has been proposed to have a crucial impact on the stabilization of the positively charged iminium transition state.^2^, ^7^, ^23^ This is supported by the 50 % decrease in specific activity of the SDR‐*Zt* E212A variant, as well as the overall negative potential at the substrate‐binding site of IREDs and of the K→D βHAD variants (except for the positively charged carboxyl‐binding site).^2^, ^37^, ^38^

The broad spectrum of accepted substrates by IREDs^20^, ^21^, ^39^, ^40^ indicates that pure lock and key models are not adequate to explain enzyme‐catalyzed imine reduction. Recent crystallographic studies report the existence of open and close conformations of IRED, βHAD, and SDR complexes as well as induced fit effects upon ligand binding to βHADs and SDRs.^16^, ^24^, ^26^, ^27^ However, the lack of co‐crystallized imine substrates limits the deduction of mechanistic principles. Moreover, dynamic aspects, such as the influence of flexible loops on substrate specificity, enantioselectivity, and promiscuous activities, have to be taken into account. This can be exemplified by noroxomaritidine reductase (SDR‐*Np*): in addition to its physiological enone reduction activity,^16^ SDR‐*Np* is able to reduce different keto and imine substrates.^17^ The crystal structure of SDR‐*Np* indicates that an intrinsically disordered loop (after β5) forms a kinked helix upon substrate binding, resulting in the catalytically active conformation. Remarkably, all investigated enzymes display substrate‐binding residues situated in helical structures involved in conformational changes or in flexible loop regions.^16^, ^24^, ^26^, ^27^ The functional relevance of dynamic fluctuations between multiple protein states has been long recognized,^41^ and is supported by recent studies in the context of promiscuous binding and unstructured protein regions.^42^, ^43^, ^44^, ^45^

The combination of intrinsically disordered binding regions, the concept of alternative proton donors, and our proposed principles for imine reduction provide a model to support the interpretation of reported puzzling observations. Despite the exchange of the conventional proton donor by alanine, the *R*‐IRED‐*Sr* D170A and *R*‐IRED‐*St* D172A variants (IRED standard position 187) were reported to maintain a considerable residual activity of 15 % and 5 %, respectively.^23^ This residual activity could be explained by the presence of alternative proton donors, for instance Y219 and Y279 in *R*‐IRED‐*Sr*, and Y221 and Y283 in *R*‐IRED‐*St* (IRED standard positions 236 and 296, respectively). Tyrosine at standard position 236 is moderately conserved in *R*‐ and *S*‐IREDs (34 % and 44 %, respectively; Table S4), while tyrosine at standard position 296 only occurs in *R*‐IREDs (28 %; Table S4). Several residues in the close environment of Y219 and Y279 in *R*‐IRED‐*Sr* could serve as flanking residues in respect to conformational changes upon substrate binding. Due to the lack of crystallized proton donor variants, no distinct flanking residues are proposed. We expect the existence of multiple proton donor positions in IREDs with adequate flanking residues for p*K*_a_ adjustment. Further to the herein proposed proton donors, amino acids in reasonable positions could be involved in catalysis, provided they result in a functional proton transfer. This is supported by the characterization of a imine reductase lacking the conventional aspartate, but displaying a histidine residue at a neighboring position.^34^ The existence of multiple alternative proton donors is further supported by the *R*‐IRED‐*Ao* Y179A and Y179F variants which maintained imine‐reducing activity.^24^ The altered enantioselectivities and kinetic parameters of these variants point toward an alternative stabilization of the transition state. Thus, we suspect that Y231 (IRED standard position 245) provides an alternative solution for proton donation as it is considerably conserved in *R*‐ and *S*‐IREDs (63 % and 13 %, respectively; Table S4), it is located on a partially unfolded helix providing a flexible substrate‐binding loop, and there are three reasonable flanking residues (M127, Q217, I218). The sc‐βHAD‐*At* K170F variant resulted in an eightfold increased amine product formation,^7^ which can be explained by the presence of D239 as alternative proton donor. The protonation catalyzed by this alternative donor might be more efficient due to the less positive binding site of the K170F variant compared to the wild type. The sc‐βHAD‐*Gm* K171D variant was reported to display a significantly lower imine‐reducing activity compared to the corresponding sc‐βHAD‐*At* K170D variant. The different topological arrangement of the flanking residue K239 (as derived from the uncomplexed crystal structure) influencing the change in substrate‐binding‐site electrostatics (the K171D variant of sc‐βHAD‐*Gm* still displays a positive charge due to K239) could explain the differences in imine‐reducing activity. The K170D/N174L sc‐βHAD‐*At* and K171D/N175L sc‐βHAD‐*Gm* variants were intended to introduce a nonpolar flanking residue as present at IRED standard position 191.^7^ No activity was determined, as the variants did not result in stable protein. Based on our model, we proposed to additionally knock out the alternative proton donor to prevent repulsion. Indeed, the K170D/N174L/D239A sc‐βHAD‐*At* variant resulted in stable protein, converting 2‐methyl‐1‐pyrroline, 3,4‐dihydroisoquinoline, and 6‐phenyl‐2,3,4,5‐tetrahydropyridine with specific activities (qualitatively determined by NADPH‐depletion assay with lysate) of 34.5±1.2 mU/mL, 33.2±0.5 mU/mL, and 35.4±0.9 mU/mL. The K170D/D239A sc‐βHAD‐*At* variant showed no activity.^46^ Clearly, catalytic machinery alone is not sufficient: the substrate also has to have access to the catalytic machinery. Bulky residues in substrate access tunnels or on flexible substrate‐binding structures can impede the entrance or the productive orientation of nonphysiological substrates, respectively.^47^, ^48^ Residues with such functionalities have been described as “gatekeepers” for various enzyme families,^7^, ^49^, ^50^, ^51^ such as F231 in sc‐βHAD‐*At*, and might also play a role in classical SDRs: in SDR‐*Zt*, the substrate‐binding site in the closed conformation is narrowed by F202 on the disordered loop/helix region (after β5). This could hinder the efficient orientation and stabilization of the substrate toward the catalytic machinery. Consequently, the F202A variant results in a tenfold increased activity toward the bulky substrate TMI.

## Conclusion

In recent years, enzymes with imine‐reducing activity have increasingly been discovered in multiple enzyme families with different global sequences and structures. This implies that imine‐reducing activity is independent of homology and suggests the existence of yet hidden commonalities in imine‐reducing enzymes. The systematic structure‐based analysis of global and local features in imine‐reducing IREDs, βHADs, and SDRs has revealed common principles in the different enzyme families, thus partly rationalizing their shared catalytic scope. As these principles might also apply to other NAD(P)H‐dependent oxidoreductases, our model could support enzyme discovery, add to the diversity of imine‐reducing enzymes, and contribute to rational enzyme engineering approaches toward imine reduction.^52^

## Conflict of interest

The authors declare no conflict of interest.

## Supporting information

As a service to our authors and readers, this journal provides supporting information supplied by the authors. Such materials are peer reviewed and may be re‐organized for online delivery, but are not copy‐edited or typeset. Technical support issues arising from supporting information (other than missing files) should be addressed to the authors.

Supplementary
