# Supplementary material for: Systematic Evaluation of Imine‐Reducing Enzymes: Common Principles in Imine Reductases, β‐Hydroxy Acid Dehydrogenases, and Short‐Chain Dehydrogenases/ Reductases
Source: Chembiochem. 2020 May 29;21(18):2689–95. doi: 10.1002/cbic.202000213 (PMC7540600; doi:10.1002/cbic.202000213)
Supplement: Supplementary file 1 — Supplementary [file CBIC-21-2689-s001.pdf]

ChemBioChem

Supporting Information

Systematic Evaluation of Imine-Reducing Enzymes: Common Principles in Imine Reductases, β-Hydroxy Acid Dehydrogenases, and Short-Chain Dehydrogenases/Reductases

Peter Stockinger, Sebastian Roth, Michael Müller, and Jürgen Pleiss*© 2020 The Authors. Published by Wiley-VCH Verlag GmbH & Co. KGaA.
This is an open access article under the terms of the Creative Commons Attribution License, which permits use, distribution and reproduction in any medium, provided the original work is properly cited.

I.	Materials and Methods	2-6
II.	Detailed comparison of substrate-binding sites	7
III.	Supporting Figures	8-18
IV.	Supporting Tables	19-23
V.	References	24

I. Material and Methods

(A) For the structural comparison of IREDs, βHADs, and SDRs, representative proteins from each family were chosen (Table 3): the IRED from *S. roseum* (*R*-IRED-*Sr*, PDB: 5OCM), the short-chain glyoxylate reductase from *A. thaliana* (sc-βHAD-*At*, PDB: 3DOJ), the long-chain 6-phosphogluconate dehydrogenase from *L. lactis* (lc-βHAD-*Ll*, PDB: 2IYP), the dimeric Classical SDR from *Burkholderia vietnamiensis* (SDR-*Bv*, PDB: 5IDW), and the tetrameric Classical SDR noroxomaritidine reductase from *N. pseudonarcissus* (SDR-*Np*, PDB: 5FFF).

(B) To identify the common principles for an enzyme to mediate imine reduction, the substrate-binding sites and catalytic sites of eight imine-reducing enzymes were compared (Table S1): three IREDs from *S. roseum* (*R*-IRED-*Sr*, PDB: 5OCM), *A. orientalis* HCCB10007 (*R*-IRED-*Ao*, PDB: 5FWN), and *Bacillus cereus* (*S*-IRED-*Bc*, PDB: 4D3F); three βHADs from *A. thaliana* (sc-βHAD-*At*, PDB: 3DOJ), *L. lactis* (lc-βHAD-*Ll*, PDB: 2IYP), and *G. metallireducens* (sc-βHAD-*Gm*, PDB: 3PEF); and two Classical SDRs from *N. pseudonarcissus* (SDR-*Np*, PDB: 5FFF) and *Z. treatiae* (SDR-*Zt*, PDB: 6Y4D). The NAD(P)H-binding domains of IREDs, βHADs, and SDRs have been classified as NAD(P)H-binding Rossmann-like domains3 (domain 3.40.50.720); therefore, the respective domains of the crystal structures of *R*-IRED-*Sr* (position 1–163), lc-βHAD-*Ll* (position 1–177), sc-βHAD-*At* (position 1–161), SDR-*Bv* (complete monomer), and SDR-*Np* (complete monomer) were superimposed using PyMOL.[1]

Table S1. Enzymes used for the comparison of structures and substrate-binding sites of IREDs, βHADs, and SDRs: classification, characterization, natural origin, and protein data base accession number. SDR-Bv was only used as a dimeric representative for the structural comparison, as only imine-reducing enzymes were considered for the comparison of substrate-binding sites.

Family	Description	Host organism	Accession	ID
**IRED**	classified as *R*-selective IRED	*Streptosporangium roseum*	5OCM	*R*-IRED-*Sr*
**IRED**	classified as *R*-selective IRED	*Amycolatopsis orientalis* HCCB10007	5FWN	*R*-IRED-*Ao*
**IRED**	classified as *S*-selective IRED	*Bacillus cereus*	4D3F	*S*-IRED-*Bc*
**βHAD**	short-chain glyoxylate reductase	*Arabidopsis thaliana*	3DOJ	sc-βHAD-*At*
**βHAD**	long-chain 6-phosphogluconate dehydrogenase	*Lactococcus lactis*	2IYP	lc-βHAD-*Ll*
**βHAD**	short-chain γ-hydroxybutyrate dehydrogenase	*Geobacter metallireducens*	3PEF	sc-βHAD-*Gm*
**SDR**	dimeric Classical SDR	*Burkholderia vietnamiensis*	5IDW	SDR-*Bv*
**SDR**	tetrameric Classical SDR; noroxomaritidine reductase	*Narcissus pseudonarcissus*	5FFF	SDR-*Np*
**SDR**	tetrameric Classical SDR	*Zephyranthes treatiae*	6Y4D	SDR-*Zt*

(C) For each of the protein families, the BioCatNet database system provides a standard numbering scheme to enable the comparison of all protein family members. The numbering schemes are generated using a profile hidden Markov model (HMM), which is trained by a multiple sequence alignment of representative proteins. Subsequently, for each family member a standard number is assigned to each amino acid position by aligning the sequence to the profile HMM and transferring the position number from a reference sequence.[53] For IREDs, the reference protein is *R*-IRED-*Sk* from Streptomyces kanamyceticus (PDB: 3ZHB).[2,23] For SDRs, separate numbering schemes were generated for Classical SDRs (reference: galactitol dehydrogenase from Rhodobacter sphaeroides, PDB: 2WDZ) and Extended SDRs (reference: dTDP-glucose 4,6-dehydratase from Streptomyces venezuelae, PDB: 1R6D).[19] Due to the significant difference in length of the long- and short-chain βHADs, no standard numbering was applied in these cases, but the equivalent positions were determined by superimposing the according structures using PyMOL.[1] The conservation analysis of *R*-IREDs, *S*-IREDs was derived from the respective database (IRED v3 and SDRED v2.2.3) of the BioCatNet framework. To generate this, a multiple sequence alignment was done while the according standard numbering scheme was applied. Determination of the percentage of each amino acid for every alignment column resulted in a conservation table for all standard positions.

(D) To calculate the distribution of the electrostatic potential in the substrate-binding site from the crystal structures, the PDB2PQR server (Version 2.0.0) utilizing PROPKA[2,3] was used with the PARSE force field and a pH of 7 to calculate the pKa and predict the protonation state of titratable side chains. Substrate-binding-site electrostatics were calculated and visualized by the ABPS Plug-In in PyMOL[1,4,5] using the PQR output file generated by PDB2PQR. Only enzyme–ligand complexes were chosen; therefore, *S*-IRED-*Bc* and sc-βHAD-*At* were not considered. To investigate the differences in imine conversion of the βHAD K→D variants, substrate-binding-site electrostatics were also determined for lc-βHAD-*Ll* K184D and for sc-βHAD-*Gm* K171D. Point mutations were introduced via the PyMOL mutagenesis tool.

(E) Alanine variants of selected residues in the substrate-binding site of SDR-Zt were generated to evaluate their importance to imine reduction. In the case of C150, mutations C150A, C150S, and C150D were introduced. pET28a_Zt_SDR served as the template for the creation of variants on the basis of the QuikChange site-directed mutagenesis protocol.[6] PCR was performed using a Thermo Scientific Phusion Flash High-Fidelity PCR Master Mix according to the manufacturer's protocol with the corresponding primers (Table S2). After digestion with DpnI (New England BioLabs) and transformation of E. coli DH5α cells, the sequences were verified by Sanger sequencing (GATC/eurofins Genomics).

(F) Enzyme production and purification were performed as described previously[17] except that the expression temperature was raised to 22 °C. SDS-PAGE of the purified protein was performed to evaluate the success of the purification process (Figure S1).

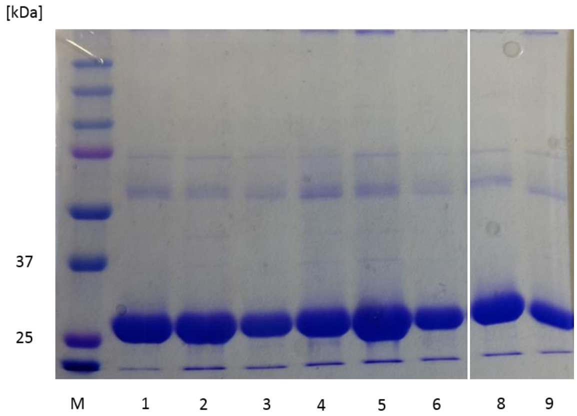

Figure S1. SDS-PAGE of purified SDR-Zt (1) and variants Y100A (2), N102A (3), C149A (4), C150A (5), H158A (6), F202A (8), E212A (9) with a Precision Plus Protein Dual Color Standards (Biorad) as marker (M). Line 7 was removed, as it represented a variant which was not considered in this work.

The specific activities of SDR-*Zt* and its variants were determined by monitoring the decrease in absorbance of the cofactor NADPH at 340 nm with a V-730 spectrophotometer (Jasco International) equipped with a temperature-controlled PSC-718 cell holder. The assays were performed on a 1 mL scale at 30 °C. For each assay, 970 µL MOPS buffer (100 mM, pH 6.5) was mixed with 10 µL NADPH (from a 25 mM stock solution in MOPS buffer, pH 6.5) and 10 µL 2,3,3-trimethylindolenine (TMI) (from a 100 mM stock solution in DMSO). The reactions were started by adding 10 µL of the purified enzyme solution (resulting enzyme concentration: 0.042–0.069 mg/mL). All reactions were performed in triplicate. In addition, blank experiments without enzyme were performed. The assays were measured over a time period of 15 min every 22 sec. Slopes were determined with Spectra Manager Version 2.12.00 (Jasco) based on time periods that comprised at least 20 data points within the linear range of absorbance decrease. Slopes were corrected by the rate of spontaneous absorbance decrease obtained from the respective blank experiment. In vitro experiments to determine the conversion of TMI on an analytical scale (250 µL) were performed in MOPS buffer (100 mM, pH 6.5) with purified enzyme at 30 °C for 3 h. Each assay contained 1.0 mg/mL purified enzyme, 10 mM substrate (added from a 1 M stock solution in DMSO), 20 mM glucose, 0.25 mg/mL glucose dehydrogenase (*Bacillus subtilis*), and 0.5 mM $NADP^+$. All assays were performed in triplicate, stopped by adding 15 µL NaOH (8 M), and extracted with 500 µL ethyl acetate. After centrifugation (10 min, 14000 rpm), the extract was used for chiral-phase HPLC analysis to determine conversion and enantiomeric excess (Table S3, Figure S2, Figure S3).

Table S2. Primers for creation of SDR-*Zt* variants:

Variant	Forward primer (5´→3´)	Reverse primer (5´→3´)
**Y100A**	CAATGCCGGTGGC**GCG**GTGAATAAACCGATTG	CAATCGGTTTATTCAC**CGC**GCCACCGGCATTG
**N102A**	GGTGGCTATGTG**GCC**AAACCGATTGATGATGTTAC	GTAACATCATCAATCGGTTT**GGC**CACATAGCCACC
**S148A**	CATTGTTCATGTGAGC**GCG**TGTTGTGCACAG	CTGTGCACAACA**CGC**GCTCACATGAACAATG
**C149A**	CATGTGAGCAGC**GCC**TGTGCACAGATTGCAC	GTGCAATCTGTGCACA**GGC**GCTGCTCACATG
**C150A**	GTGAGCAGCTGT**GCA**GCACAGATTGCACTG	CAGTGCAATCTGTGC**TGC**ACAGCTGCTCAC
**C150D**	GTGAGCAGCTGT**GAT**GCACAGATTGCACTGCCTGG	CCAGGCAGTGCAATCTGTGC**ATC**ACAGCTGCTCAC
**C150S**	GTGAGCAGCTGT**TCT**GCACAGATTGCACTGCCTGG	CCAGGCAGTGCAATCTGTGC**AGA**ACAGCTGCTCAC
**H158A**	GCACTGCCTGGT**GCC**AGCATGTATAGCGCAACC	GGTTGCGCTATACATGCT**GGC**ACCAGGCAGTGC
**Y161A**	CTGGTCATAGCATG**GCG**AGCGCAACCAAAG	CTTTGGTTGCGCT**CGC**CATGCTATGACCAG
**K165A**	ATGTATAGCGCAACC**GCG**GGTGCAATTAATCAGC	GCTGATTAATTGCACC**CGC**GGTTGCGCTATAC
**F202A**	GAGCAGCGAACCG**GCC**GTTAATGATAAAGATGC	GCATCTTTATCATTAAC**GGC**CGGTTCGCTGCTC
**E212A**	GATGCAGTTGCCAAA**GCA**GTTGCACGCGTTCC	GGAACGCGTGCAACT**GCT**TTGGCAACTGCATC

Chiral-phase HPLC analysis was performed on an HP 1100 chromatography system (Agilent Technologies) using a Chiralcel OD-H column (250 mm × 4.6 mm; Daicel Inc., West Chester, USA). The HPLC method was changed during the course of the investigations. Therefore, two different HPLC methods are listed (Table S2).

Table S3. Methods used for HPLC analysis

Method	Flow rate in [mL·min^{-1}]	Mobile phase
A	0.7 mL·min^{-1}, 40° C	n-hexane/2-propanol, 99:1
B	0.6 mL·min^{-1}, 30°C	n-hexane/2-propanol/diethylamine, 98:2:0.1

The resulting chromatograms were used for the determination of conversion and enantiomeric excess. The different relative responses of imine TMI and the respective amine product at the detection wavelength of 254 nm were corrected by the experimentally determined response factor obtained from standard curves (A_{254nm}imine/A_{254nm}amine=1.8). The absolute configuration of the products was assigned according to the literature.[7]

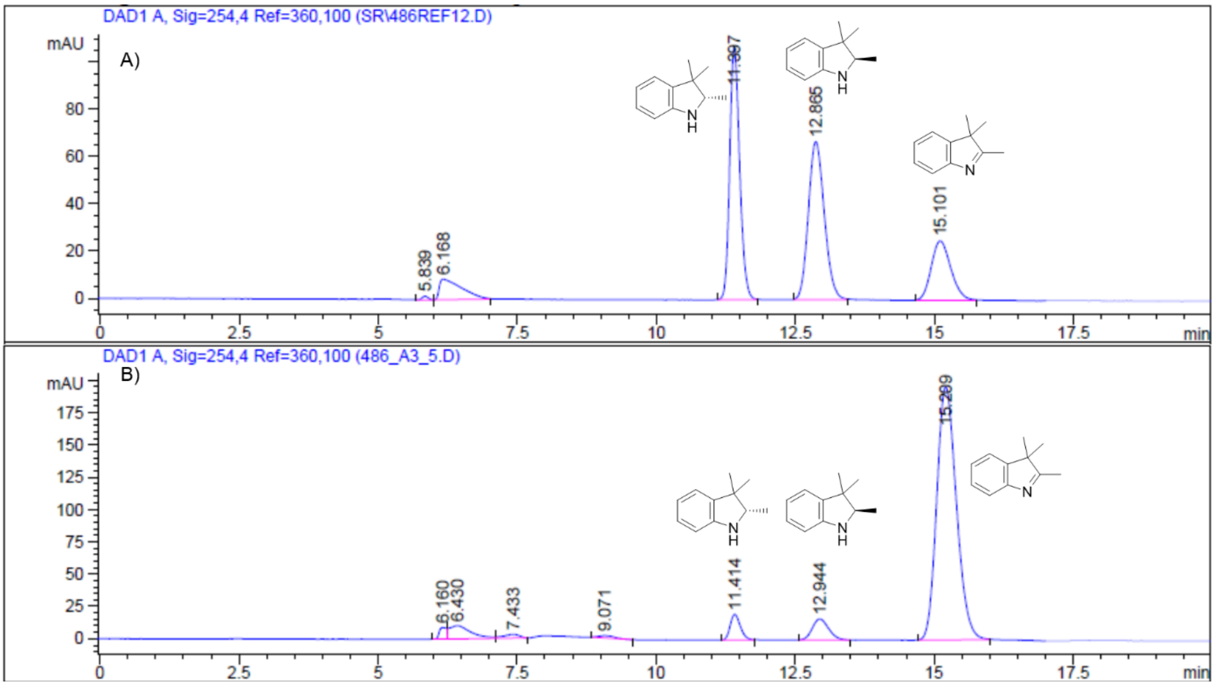

Figure S2. Chiral-phase HPLC chromatograms to determine conversion and resulting enantiomeric excess of product using method A; chromatogram A: mixture of substrate and racemic product reference ($NaBH_4$ reduction); chromatogram B: enzymatic reduction catalyzed by SDR-*Zt*_S148A.

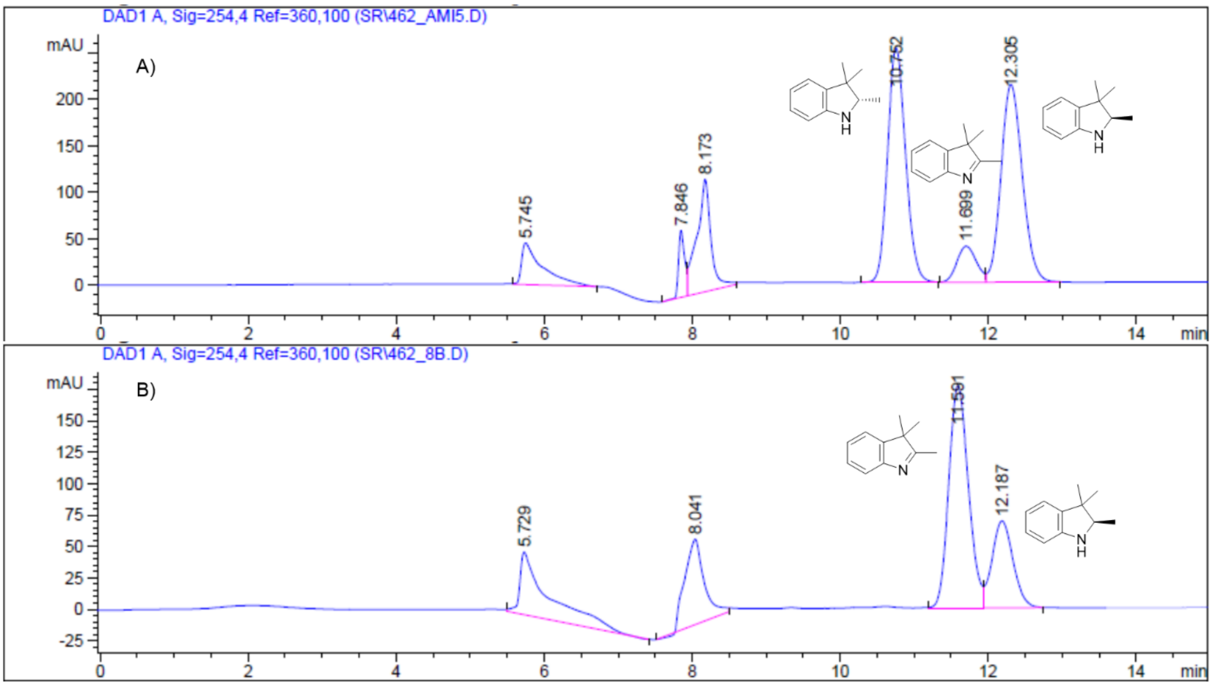

Figure S3. Chiral-phase HPLC chromatograms to determine conversion and resulting enantiomeric excess of product using method B; chromatogram A: mixture of substrate and racemic product reference ($NaBH_4$ reduction); chromatogram B: enzymatic reduction catalyzed by SDR-Zt.

II. Detailed comparison of substrate-binding sites

The substrate-binding site of IREDs is mainly composed of nonpolar amino acids (IRED standard positions 136, 137, 139, 191, 194, 195, 198, 225, 254, 259, 296).

In *R*-IREDs, four nonpolar positions (IRED standard positions M137, L191, M194, W225) are highly conserved (>75%, Table S4). Six positions (IRED standard positions 139, 195, 232, 236, 255, 296) diverge in their polarity. Additionally, the polar character of standard positions 111 (45% T, 33% S, 20% N) and 261 (31% H, 25% T, 19% N) is conserved.

In *S*-IREDs, three nonpolar positions (IRED standard positions V136, P139, F194) are highly conserved (>88%, Table S4). Eight positions (IRED standard positions 137, 191, 195, 225, 232, 236, 255, 296) diverge in their polarity and, moreover, two polar residues (IRED standard positions S111, H261) are conserved (100%).

The substrate-binding site of lc-βHAD-*Ll* is composed of six nonpolar residues (M141, V183, M185, M195, I367, F450) (Table S5) and eight polar residues (N102, S128, H181, H187, N188, T192, C366, H453). The substrate-binding site of sc-βHAD-*At* is composed of nine nonpolar residues (L135, M169, L171, V173, M177, M214, F231, M240, F277) (Table S5) and five polar residues (T95, S121, N174, H235, Q236). The substrate-binding site of sc-βHAD-*Gm* is composed of 10 nonpolar residues (L136, M170, L172, V147, M178, M215, F232, M237, L241, F278) (Table S5) and four polar residues (T96, S122, N175, H236).

In comparison to IREDs, more positively charged residues were found in βHADs which contribute to the binding site of the carboxylic acid moiety.[2,28,29] Several residues with a similar structural location and chemical functionality did not match with the exact position in a sequence-based alignment which might indicate alternative functional units. In lc-βHAD-*Ll*, for example, nonpolar (I367, F450) and positively charged residues (R289, R447), which do not appear in the shorter sc-βHAD-*At* and sc-βHAD-*Gm*, are equivalently present in alternative regions for substrate binding upstream in sequence (Table S5). Interestingly, one acidic residue is present in all considered βHAD substrate-binding sites (lc-βHAD-*Ll*: E191, sc-βHAD-*At*: D239, sc-βHAD-*Gm*: D240).

The substrate-binding site of the investigated Classical SDRs is composed of four nonpolar (Classical SDR standard positions Y96, I155, Y159, F200) and eight polar residues (Classical SDR standard positions N98, S144, C145, C146, H156, K163, S197, E210). In Classical SDRs, the four nonpolar positions display no conservation (Table S6). Except for the catalytic residues (Classical SDR standard positions S144, Y159, K163), the polar residues also show no conservation and, moreover, the occurrence of Y96, N98, C145, C146, I155, H156, F200, and E210 is very rare (<4%, Table S6).

III. Supporting Figures

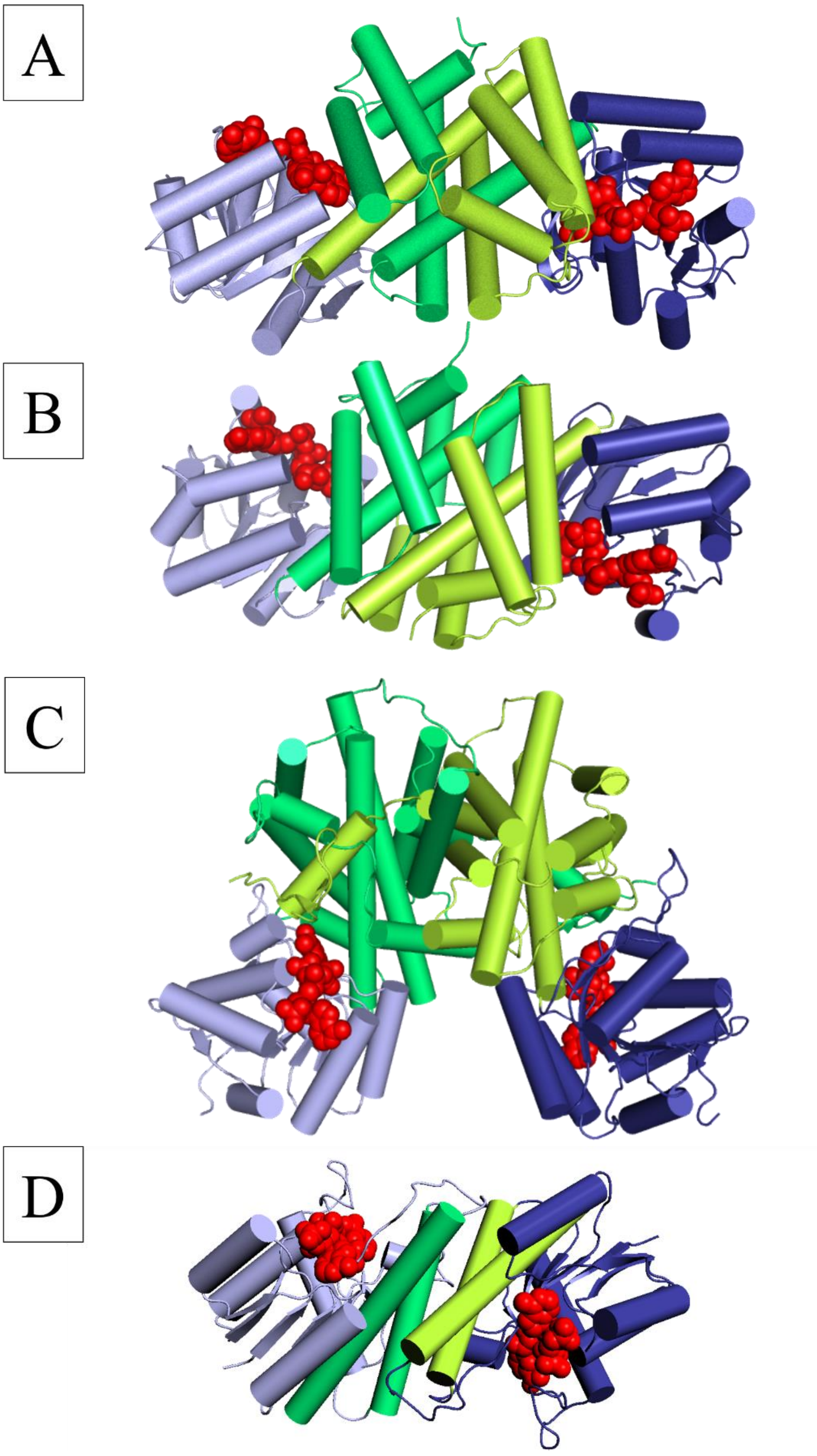

Figure S4. Architecture of dimeric reductases. (A) R-IRED-Sr structure 5OCM. (B) sc-βHAD-At structure 3DOJ. (C) lc-βHAD-Ll structure 2IYP. (D) SDR-Bv structure 5IDW. The bound NADPHs are displayed as red spheres while the Rossmann-like NADPH-binding domains are shown in light blue (monomer 1) and dark blue (monomer 2). The helical structures providing the contact space for dimerization are colored in different green shadings to display the affiliation to the respective monomer (dark green: monomer 1; light green: monomer 2).

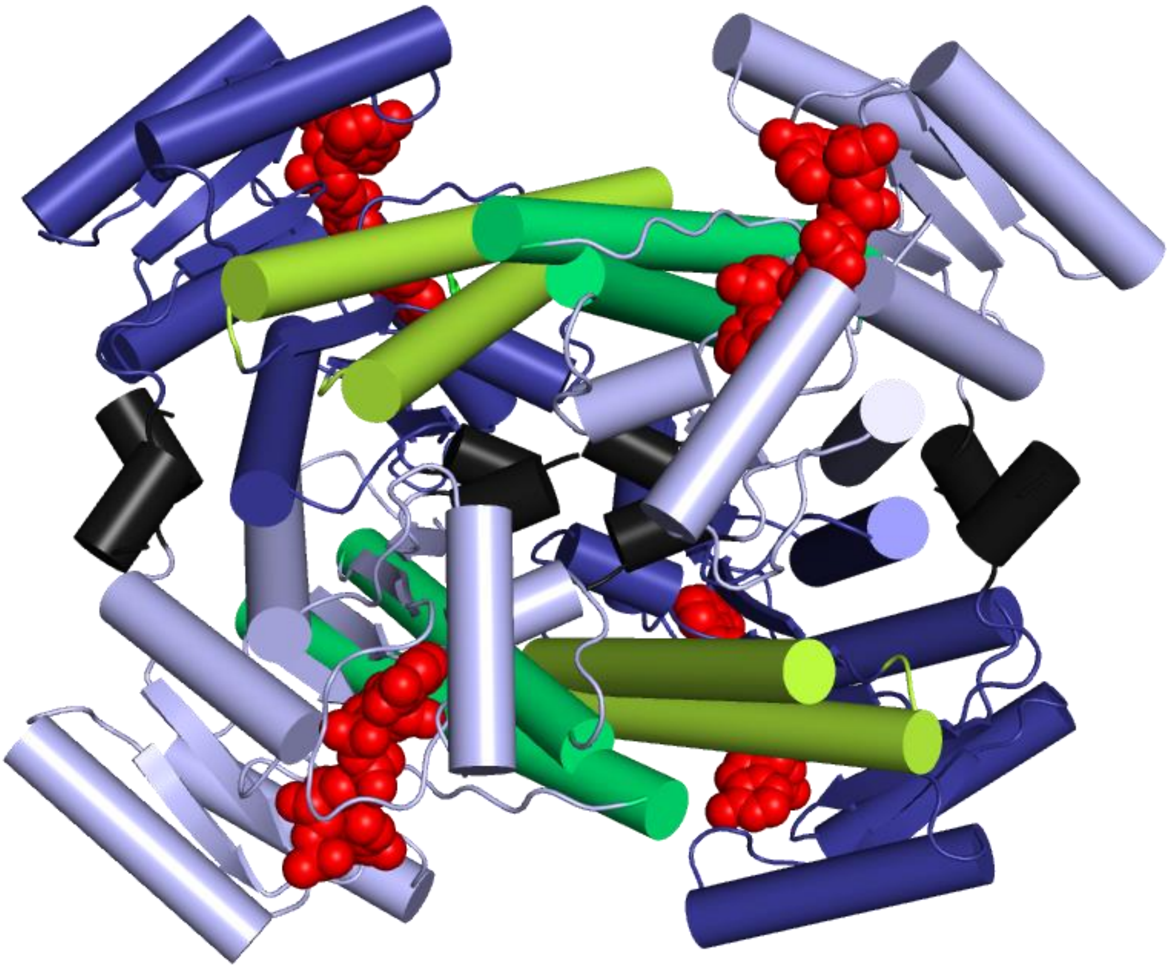

Figure S5: Architecture of the tetrameric SDR-*Np*. The long helices forming connecting the dimer are colored dark green (monomer 1) and light green (monomer 2) while the remaining Rossmann fold is colored light blue (monomer 1) and dark blue (monomer 2). The bound NADPHs are displayed as red spheres. The additional terminal helices stabilizing the tetrameric assembly of the two dimers, are shown in black.

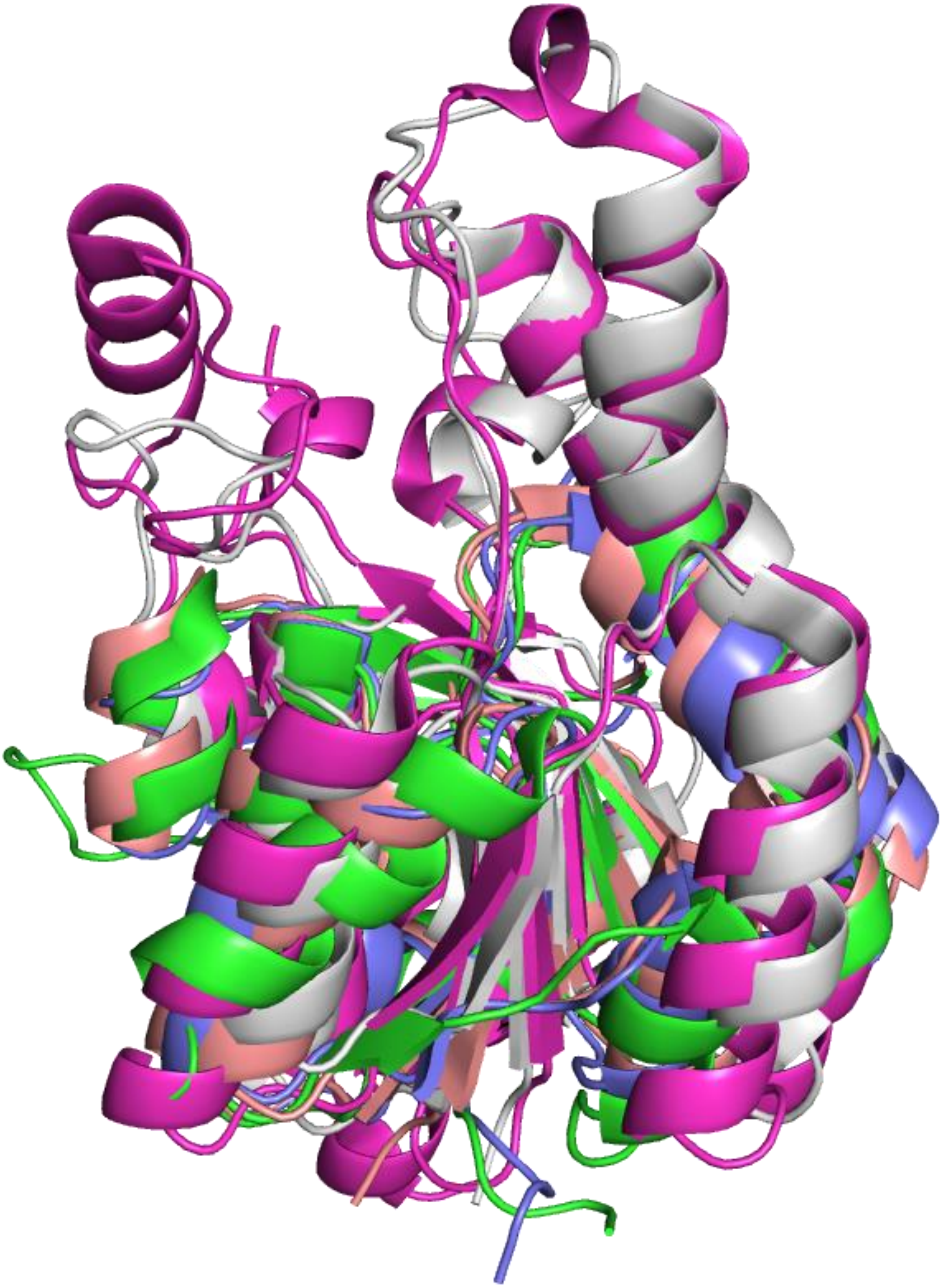

Figure S6: Superposition of the Rossmann-like NAD(P)H-binding domains of *R*-IRED-*Sr* (blue), lc-βHAD-*Ll* (green), sc-βHAD-*At* (salmon), SDR-*Bv* (light grey) and SDR-*Np* (pink).

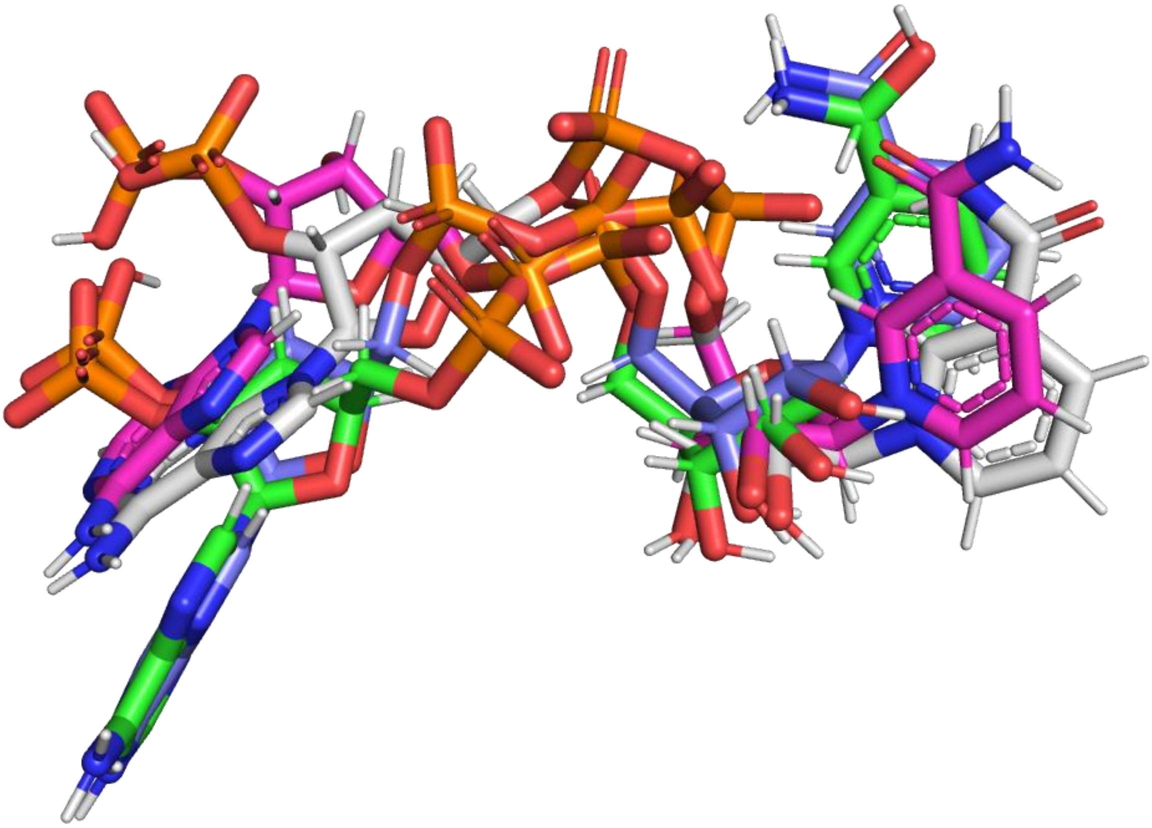

Figure S7: Similar orientation of the bound NADPH derived from the superposition of the Rossmann-like domains of *R*-IRED-*Sr* (blue), lc-βHAD-*Ll* (green), SDR-*Bv* (light grey) and SDR-*Np* (pink).

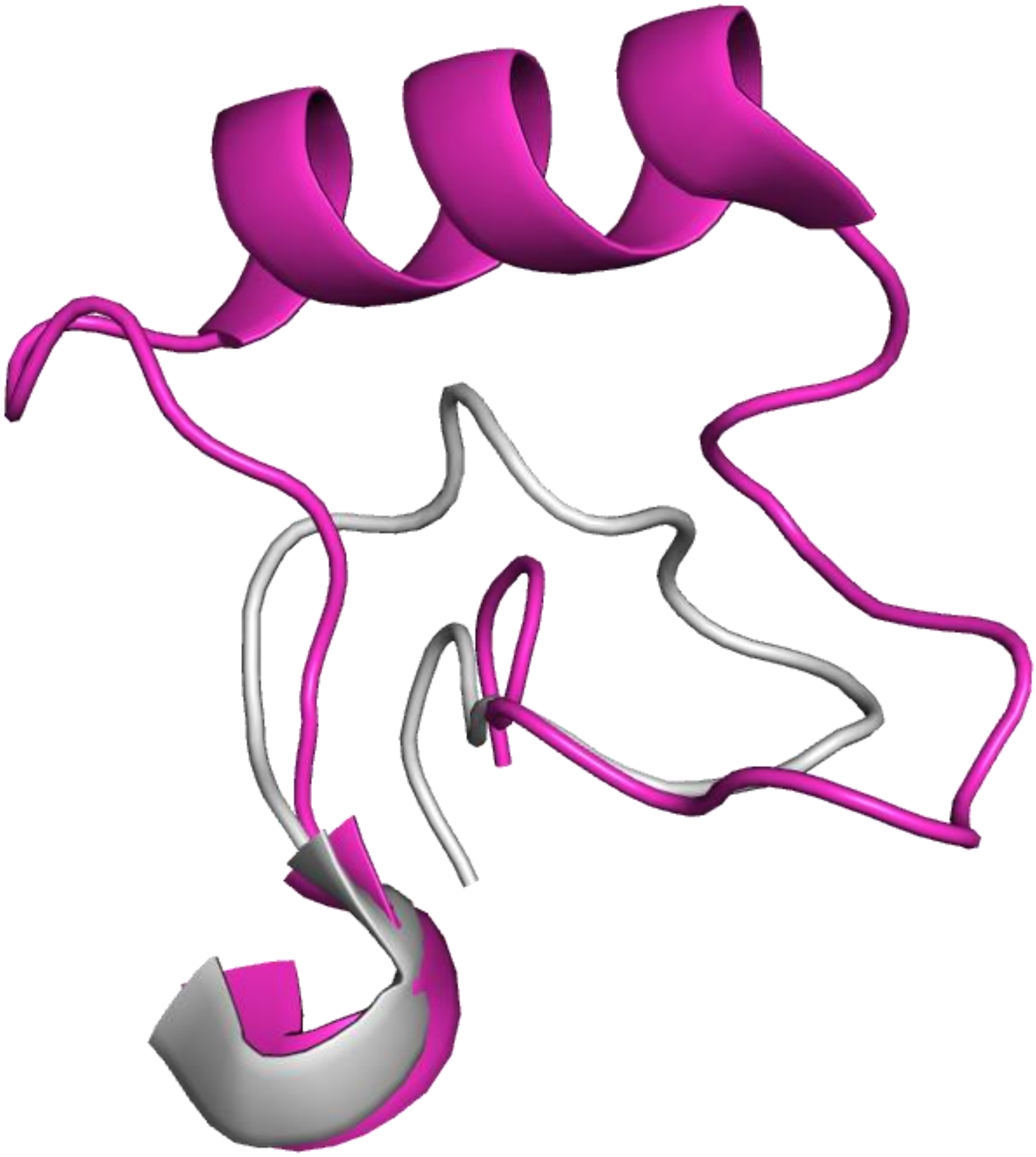

Figure S8: Classical SDR substrate binding loop involved in conformational changes after substrate binding, varying in SDR-*Bv* (P174-P190; light grey) and imine reducing SDR-*Np* (P205-P238; pink).

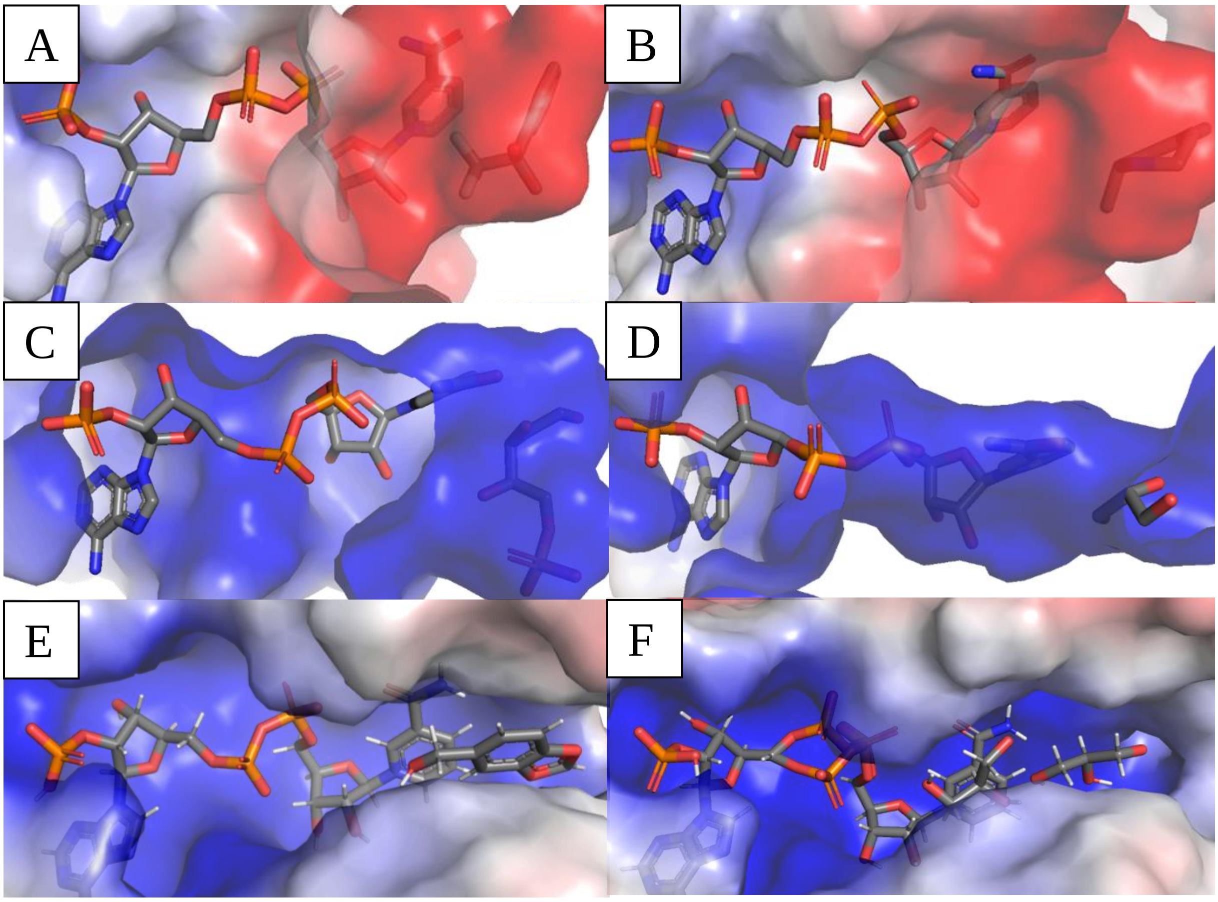

Figure S9: Visualization of substrate binding site electrostatics exemplary shown for *R*-IRED-*Sr* (A), *R*-IRED-*Ao* (B), lc-βHAD-*Ll* (C), sc-βHAD-*Gm* (D), SDR-*Np* (E) and SDR-*Zt* (F) complexed with NADPH and different ligands. While the blue coloring represents positively charged regions, negative charges are shown in red. To represent the substrate binding site electrostatics of these closed conformations clearly, a surface transparency setting of 20 % was applied.

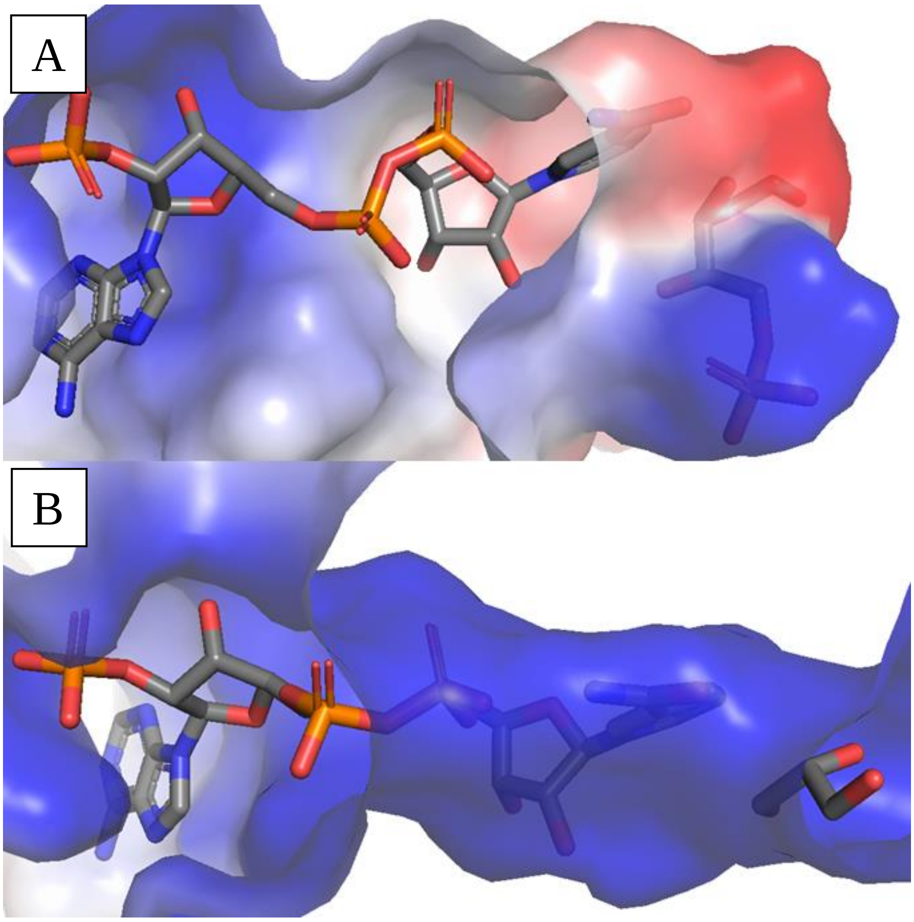

Figure S10: Visualization of substrate binding site electrostatics shown for lc-βHAD-*Ll* K→D mutant (A) and sc-βHAD-*Gm* K→D mutant (B) complexed with NADPH and the according ligands. While the blue coloring represents positively charged regions, negative charges are shown in red. To represent the substrate binding site electrostatics of these closed conformations clearly, a surface transparency setting of 20 % was applied.

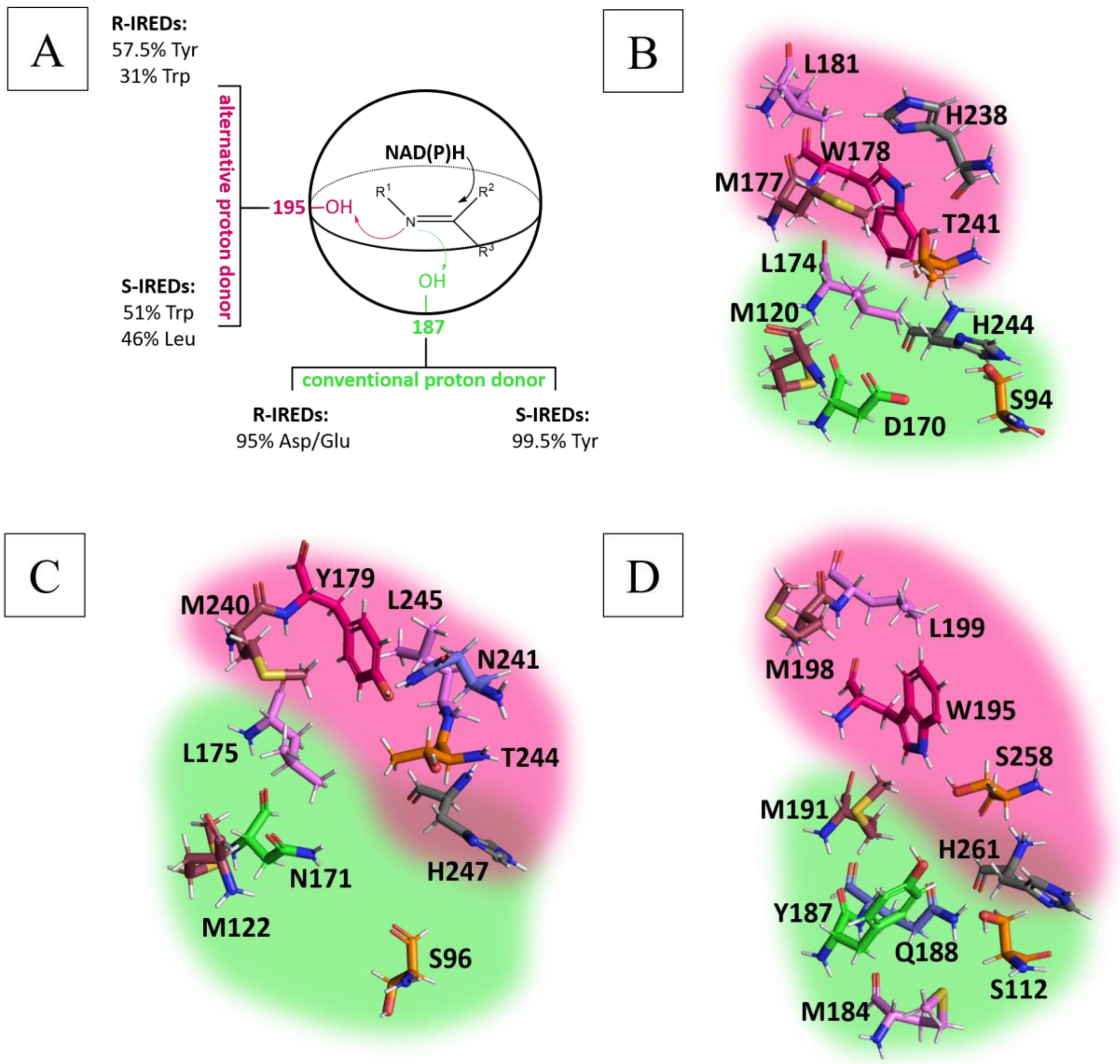

Figure S11: (A) Scheme of IRED substrate binding site. Next to the conventional proton donor on standard position 187 (green), the alternative proton donor position 195 is proposed (rosy). Additionally, the relative occurrence of those residues in both, *R*- and *S*-IREDs, is shown. These proton donors including their proposed flanking residues are shown for *R*-IRED-*Sr* (B), *R*-IRED-*Ao* (C), and *S*-IRED-*Bc* (D). The coloring of the flanking residues refers to the general catalytic site scheme (Figure 2A). The green amino acid and the green background shading mark the residue on conventional proton donor position with its flanking residues, while the rosy amino acid and the rosy background shading mark the residue on alternative proton donor position.

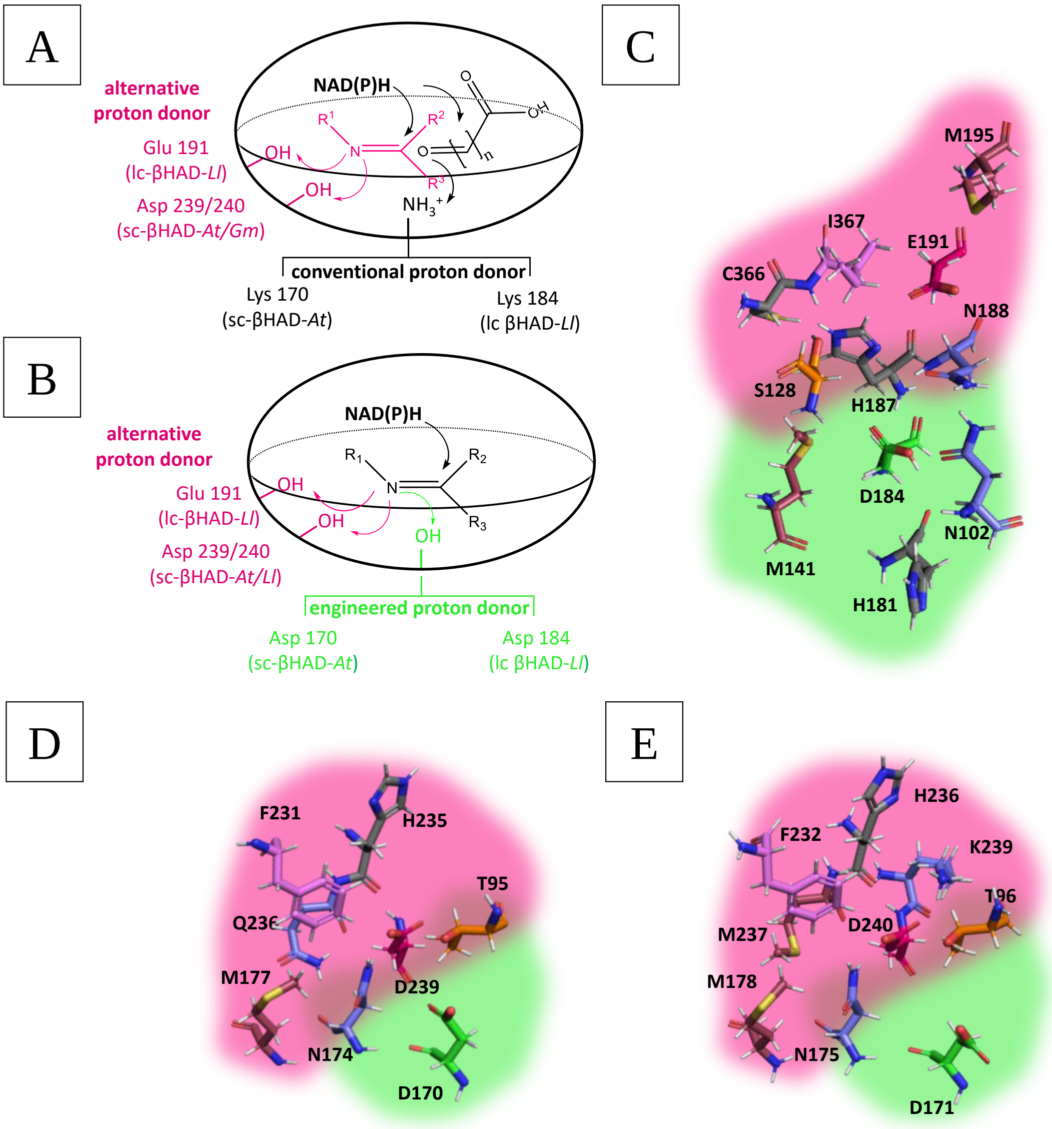

Figure S12: (A) Scheme of βHAD substrate binding site. Next to the conventional proton donor for carbonyl reduction Lys 170/171/184 (black), alternative proton donors Glu 195 (lc-βHAD-Ll), Asp 239 (sc-βHAD-*At*), and Asp 240 (sc-βHAD-*Gm*) (rosy) for imine reduction are proposed. (B) Engineered proton donors for imine reduction Asp 170/171/184 (green) and proposed alternative proton donors Glu 195 (lc-βHAD-*Ll*), Asp 239 (sc-βHAD-*At*), and Asp 240 (sc-βHAD-*Gm*) (rosy). These proton donors including their proposed flanking residues are shown for lc-βHAD-*Ll* (C), sc-βHAD-*At* (D), and sc-βHAD-*Gm* (E). The coloring of the flanking residues refers to the general catalytic site scheme (Figure 2A). The green amino acid and the green background shading mark the residue on conventional proton donor position with its flanking residues while the rosy amino acid and the rosy background shading mark the residue on alternative proton donor position.

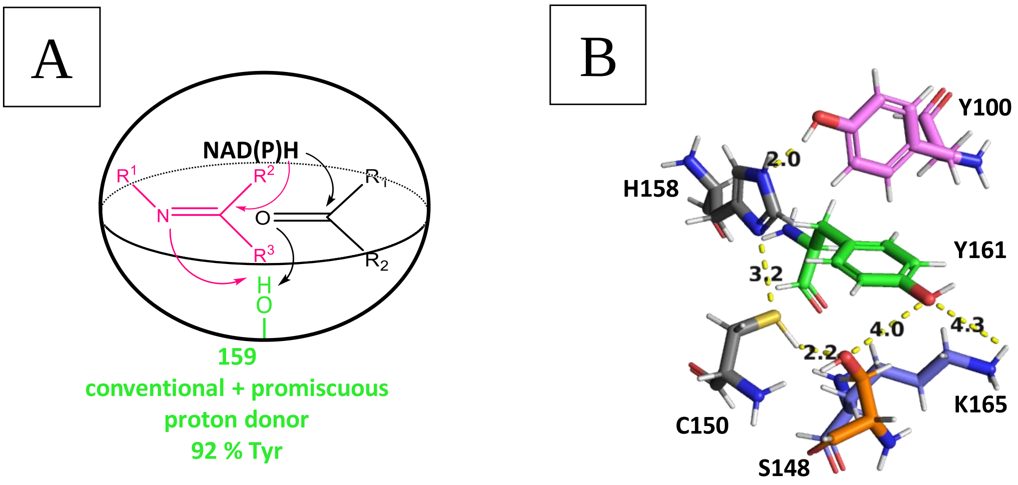

Figure S13: (A) Scheme of SDR substrate binding site. The conventional proton donor on standard position 159 (green) is also proposed to catalyze the proton donation in the promiscuous imine reduction which is marked with rosy lines. Additionally, its' relative occurrence in all Classical SDRs is shown. (B) The proton donors including their proposed flanking residues are shown for SDR-*Np*. The coloring of the flanking residues refers to the general catalytic site scheme (Figure 2A). The green amino acid marks the residue on conventional proton donor position.

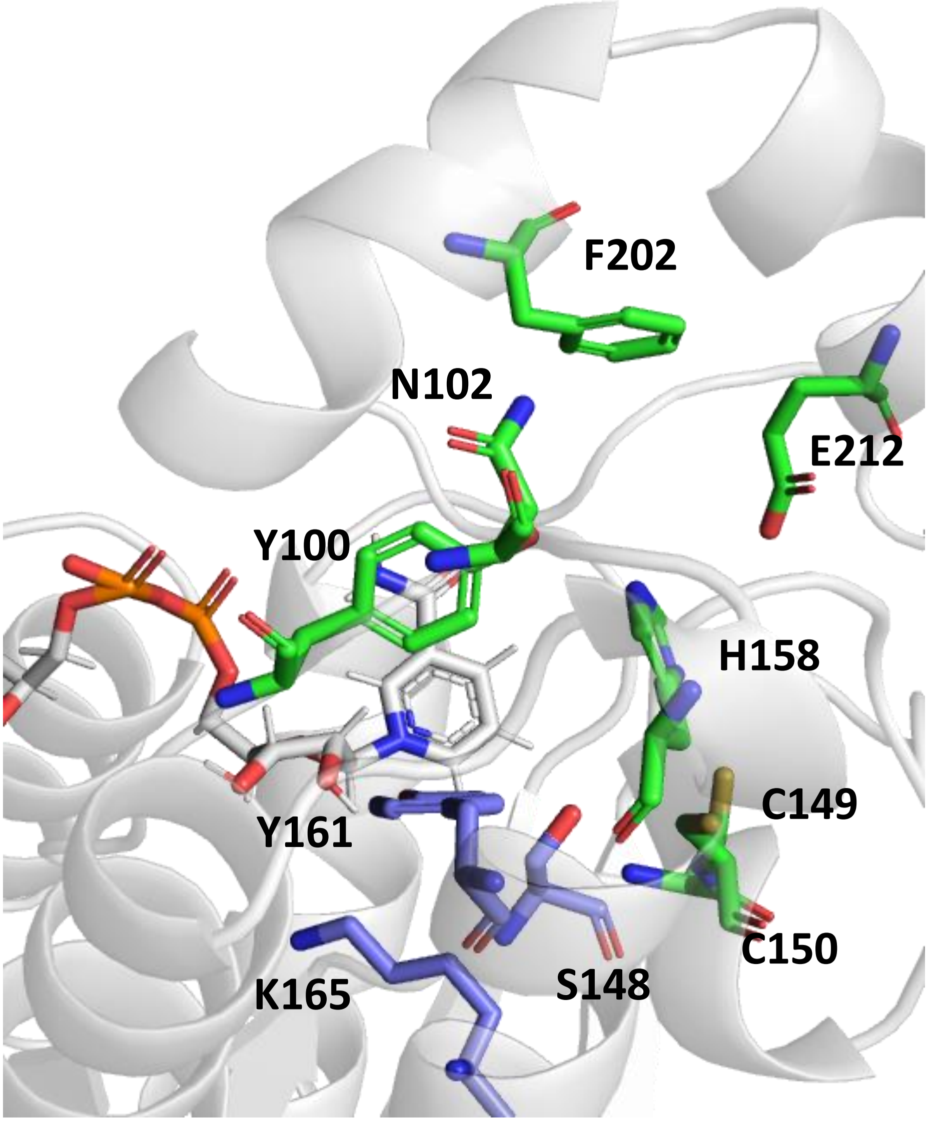

Figure S14: Substrate-binding site of SDR-*Zt*. The catalytic triad (blue) and the substrate-binding residues with rare occurrence in the family of Classical SDRs (green) selected for alanine scanning are shown.

IV. Supplementary Tables

Table S4: Equivalent residues in the substrate binding sites of *R*-IRED-*Sr*, *R*-IRED-*Ao* and *S*-IRED-*Bc* with the according standard position in the imine reductase engineering data base. Additionally, the percentual occurrence (>10 %) in the superfamily of *R*- and *S*-IREDs is shown. In the case of conventional proton donor position 187, all occurring amino acids are shown.

IRED s.p.	111	136	137	139	184	187	188	191	194	195	198	225	232	236	245	254	255	258	259	261	296
R-IRED-*Sr*	S94	I119	M120	I122	S167	D170	V171	L174	M177	W178	L181	W208	F215	Y219	Y228	T237	H238	T241	V242	H244	Y279
R-IRED-*Ao*	S96	I121	M122	P124	V168	N171	T172	L175	M178	Y179	M182	W209	I218	E222	Y231	M240	N241	T244	L245	H247	Y282
S-IRED-*Bc*	S112	V137	Q138	P140	M184	Y187	Q188	M191	F194	W195	M198	M225	F232	Y236	H245	M240	G255	S258	V259	H261	F296
Occurrence in R-IREDs	45% T<br>33% S<br>20% N	89% I	94% M	59% V<br>20% T<br>13% I	49% A<br>35% S<br>11% P	93% D<br>4% N<br>2% E<br>1% G	41% V<br>25% L<br>10% I	96 % L	97 % M	57 % Y<br>31 % W	62% F<br>24% L	76 % W	19 % F<br>18 % A<br>13 % S	34 % Y<br>15 % T	63% Y<br>15% F	48% M<br>18% V<br>12% F<br>12% T	49 % Q<br>14 % H<br>12 % N	50% A<br>33% G	24% L<br>15% M	31 % H<br>25 % T<br>19 % N	28 % Y<br>25 % L<br>14 % F
Occurrence in S-IREDs	100% S	88% V	51% M<br>24% Q	94% P	56%Q<br>36% A	100% Y	98% Q	51 % L<br>39 % M	100 % F	51 % W<br>46 % L	63% L<br>31% M	50 % T<br>13 % I	56 % F<br>14 % G<br>13 % M	44 % Y<br>16 % P<br>13 % A	84% H<br>13% Y	100% M	48 % M<br>35 % G	53% S<br>45% T	46% A<br>24% V	100 % H	47 % W<br>31 % F<br>12 % L

Table S5: Equivalent residues in the substrate binding sites of lc-βHAD-*Ll*, sc-βHAD-*At* and sc-βHAD-*Gm* derived from a structural alignment.

**lc-βHAD-*Ll***	N102	S128	M141	H181	V183	K184	M185	H187	N188	-	E191	T192	M195	-	-	-	
**sc-βHAD-*At***	T95	S121	L135	A167	M169	K170	L171	V173	N174	M177	-	-	-	A209	M214	K218	P220
**sc-βHAD-*Gm***	T96	S122	L136	A168	M170	K171	L172	V174	N175	M178	-	-	-	A210	M215	K219	G221

**lc-βHAD-*Ll***	-	-	-	-	-	-	-	R289	C366	I367	R447	F450	H453
**sc-βHAD-*At***	F231	H235	Q236	K238	D239	M240	F277	-	-	-	-	-	-
**sc-βHAD-*Gm***	F232	H236	M237	K239	D240	L241	F278	-	-	-	-	-	-

Table S6: Equivalent residues in the substrate binding sites of SDR-*Np* and SDR-*Zt* with the according standard position in the SDR engineering data base. Additionally, the percentual occurrence (>10 %) and the occurrence of the amino acids present in SDR-*Np* and SDR-*Zt* in the superfamily of Classical SDRs is shown.

**Classical SDR s.p.**	**96**	**98**	**144**	**145**	**146**	**151**	**156**	**159**	**163**	**197**	**200**	**210**
**SDR-*Np***	Y100	N102	S148	C149	C150	I155	H158	Y161	K165	T199	F202	E212
**SDR-*Zt***	Y100	N102	S148	C149	C150	L155	H158	Y161	K165	S199	F202	E212
**Occurrence in Classical SDRs**	13% A<br>11% T<br>3% Y	13% P<br>4% D<br>3% N	90% S	24% V<br>20% I<br>1% C	35% A<br>14% S<br>1% C	12% N<br>8% L<br>5% I	11% L<br>3% H	92% Y	93% K	18% T<br>6% S	11% A<br>4% F	16% L<br>1.5% E

Table S7. Putative proton donors of all investigated imine-reducing enzymes and flanking residues proposed to enable the proton transfer to imine moieties. For IREDs and SDRs, the corresponding standard position is given. The proposed flanking residues in italics need to be treated with caution due to the lack of a complexed crystal structure.

Enzyme	Proton Donor	Standard position	Flanking residues	Standard position	Proposed property
**_R_-IRED-_Sr_<br>(conventional position)**	D170	187	S94<br>M120<br>L174<br>H244	111<br>137<br>191<br>261	imine-polarizing<br>nonpolar<br>nonpolar<br>proton-mediating
**_S_-IRED-_Sr_<br>(alternative position)**	W178[a]	195	M177<br>L181<br>H238<br>T241	194<br>198<br>255<br>258	nonpolar<br>nonpolar<br>proton-mediating<br>imine-polarizing
**_R_-IRED-_Ao_<br>(conventional position)**	N171[a]	187	S96<br>M122<br>L175<br>H247	111<br>137<br>191<br>261	imine-polarizing<br>nonpolar<br>nonpolar<br>proton-mediating
**_R_-IRED-_Ao_<br>(alternative position)**	Y179	195	M240<br>N241<br>T244<br>L245<br>H247	254<br>255<br>258<br>259<br>261	nonpolar<br>donor-polarizing<br>imine-polarizing<br>nonpolar<br>proton-mediating
**_S_-IRED-_Bc_<br>(conventional position)**	Y187	187	S112<br>M184<br>Q188<br>M191<br>H261	111<br>184<br>188<br>191<br>261	imine-polarizing<br>nonpolar<br>donor-polarizing<br>nonpolar<br>proton-mediating
**_S_-IRED-_Bc_<br>(alternative position)**	W195[a]	195	*M198*<br>*L199*<br>*S258*<br>*H261*	198<br>199<br>258<br>261	nonpolar<br>nonpolar<br>imine-polarizing<br>proton-mediating
**lc-βHAD-_Ll_ K184D<br>(conventional position)**	D184	–	N102<br>S128<br>M141<br>H181<br>H187<br>N188	–<br>–<br>–<br>–<br>–<br>–	donor-polarizing<br>imine-polarizing<br>nonpolar<br>proton-mediating<br>proton-mediating<br>donor-polarizing
**lc-βHAD-_Ll_<br>(alternative position)**	E191	–	S128<br>H187<br>N188<br>M195<br>C366<br>I367	–<br>–<br>–<br>–<br>–<br>–	imine-polarizing<br>proton-mediating<br>donor-polarizing<br>nonpolar<br>proton-mediating<br>nonpolar
**sc-βHAD-_At_ K170D<br>(conventional position)**	D170	–	*T95*<br>*M169*<br>*N174*	–<br>–	imine-polarizing<br>nonpolar<br>donor-polarizing
**sc-βHAD-_At_<br>(alternative position)**	D239	–	*T95*<br>*N174*<br>*M177*<br>*F231*<br>*H235*<br>*Q236*	–<br>–<br>–<br>–<br>–<br>–	imine-polarizing<br>donor-polarizing<br>nonpolar<br>nonpolar<br>proton-mediating<br>donor-polarizing
**sc-βHAD-_Gm_ K171D<br>(conventional position)**	D171	–	T96<br>M170<br>N175	–<br>–	imine-polarizing<br>nonpolar<br>donor-polarizing
**sc-βHAD-_Gm_<br>(alternative position)**	D240	–	T96<br>N175<br>M178<br>F232<br>H236<br>M237<br>K239	–<br>–<br>–<br>–<br>–<br>–<br>–	imine-polarizing<br>donor-polarizing<br>nonpolar<br>nonpolar<br>proton-mediating<br>nonpolar<br>donor-polarizing
**SDR-_Np_**	Y161	159	Y100<br>S148<br>C150<br>H158<br>K165	96<br>144<br>146<br>156<br>163	nonpolar<br>imine-polarizing<br>proton-mediating<br>proton-mediating<br>donor-polarizing
**SDR-_Zt_**	Y161	159	Y100<br>S148<br>C150<br>H158<br>K165	96<br>144<br>146<br>156<br>163	nonpolar<br>imine-polarizing<br>proton-mediating<br>proton-mediating<br>donor-polarizing

[a] Non-proton donating.

Table S8. Conversion of substrate TMI by SDR-*Zt* and its substrate-binding-site variants after biotransformation for 3 h (10 mM TMI), and specific activities with 1 mM TMI. Except for S148A, all variants gave *R*-product with >98% enantiomeric excess. The respective Classical SDR standard position for all mutations is indicated.

Variant	Classical SDR standard position	Conversion [%]	Specific activity [mU/mg]
**WT**		42	30 ± 1
**Y100A**	96	39	2 ± 1
**N102A**	98	39	38 ± 2
**S148A**	144	17[a]	2 ± 0.1
**C149A**	145	34	19 ± 1
**C150A**	146	<1	<1
**C150S**	146	14	n.d.[b]
**C150D**	146	49	23 ± 2
**H158A**	156	32	21 ± 1
**Y161A**	159	<1	n.d.[b]
**K165A**	163	17	n.d.[b]
**F202A**	200	>99	311 ± 2
**E212A**	210	37	14 ± 4

[a] 17% *ee* of *R*-product. [b] not detectable.

V. References

[1] Schrödinger, LLC, *The PyMOL Molecular Graphics System, Version 1.8*, **2015**.

[2] C. R. Søndergaard, M. H. M. Olsson, M. Rostkowski, J. H. Jensen, *J. Chem. Theory Comput.* **2011**, *7*, 2284–2295.

[3] M. H. M. Olsson, C. R. Søndergaard, M. Rostkowski, J. H. Jensen, *J. Chem. Theory Comput.* **2011**, *7*, 525–537.

[4] N. A. Baker, D. Sept, S. Joseph, M. J. Holst, J. A. McCammon, *Proc. Natl. Acad. Sci.* **2001**, *98*, 10037–10041.

[5] E. Jurrus, D. Engel, K. Star, K. Monson, J. Brandi, L. E. Felberg, D. H. Brookes, L. Wilson, J. Chen, K. Liles, et al., *Protein Sci.* **2018**, *27*, 112–128.

[6] R. Rapley, J. Braman, C. Papworth, A. Greener, in *Nucleic Acid Protoc. Handbook,* **2003,** *57*, 31–44.

[7] H. Li, G. X. Zhang, L. M. Li, Y. S. Ou, M. Y. Wang, C. X. Li, G. W. Zheng, J. H. Xu, *ChemCatChem* **2016**, *8,* 724.
